# Definition of the Neurotoxicity-Associated Metabolic Signature Triggered by Berberine and Other Respiratory Chain Inhibitors

**DOI:** 10.3390/antiox13010049

**Published:** 2023-12-28

**Authors:** Ilinca Suciu, Johannes Delp, Simon Gutbier, Julian Suess, Lars Henschke, Ivana Celardo, Thomas U. Mayer, Ivano Amelio, Marcel Leist

**Affiliations:** 1 In Vitro Toxicology and Biomedicine, Department Inaugurated by the Doerenkamp-Zbinden Foundation, University of Konstanz, 78464 Konstanz, Germany; 2 Graduate School of Chemical Biology, University of Konstanz, 78464 Konstanz, Germany; 3 Department of Molecular Genetics, University of Konstanz, 78464 Konstanz, Germany; 4 Division for Systems Toxicology, Department of Biology, University of Konstanz, 78464 Konstanz, Germany

**Keywords:** mitochondrial complex I, berberine, metabolomics, combi-omics, stress-response, neurotoxicity, screen hits

## Abstract

To characterize the hits from a phenotypic neurotoxicity screen, we obtained transcriptomics data for valinomycin, diethylstilbestrol, colchicine, rotenone, 1-methyl-4-phenylpyridinium (MPP), carbaryl and berberine (Ber). For all compounds, the concentration triggering neurite degeneration correlated with the onset of gene expression changes. The mechanistically diverse toxicants caused similar patterns of gene regulation: the responses were dominated by cell de-differentiation and a triggering of canonical stress response pathways driven by ATF4 and NRF2. To obtain more detailed and specific information on the modes-of-action, the effects on energy metabolism (respiration and glycolysis) were measured. Ber, rotenone and MPP inhibited the mitochondrial respiratory chain and they shared complex I as the target. This group of toxicants was further evaluated by metabolomics under experimental conditions that did not deplete ATP. Ber (204 changed metabolites) showed similar effects as MPP and rotenone. The overall metabolic situation was characterized by oxidative stress, an over-abundance of NADH (>1000% increase) and a re-routing of metabolism in order to dispose of the nitrogen resulting from increased amino acid turnover. This unique overall pattern led to the accumulation of metabolites known as biomarkers of neurodegeneration (saccharopine, aminoadipate and branched-chain ketoacids). These findings suggest that neurotoxicity of mitochondrial inhibitors may result from an ensemble of metabolic changes rather than from a simple ATP depletion. The combi-omics approach used here provided richer and more specific MoA data than the more common transcriptomics analysis alone. As Ber, a human drug and food supplement, mimicked closely the mode-of-action of known neurotoxicants, its potential hazard requires further investigation.

## 1. Introduction

Novel approach methods (NAMs) in toxicology allow screens for potential environmental toxicants with a higher throughput than conventional approaches. In addition, they offer multiple possibilities to follow up on screen hits in order to determine their toxicological relevance and to identify modes of action relevant to the toxicity in target cells, tissues, organs and organisms. Such studies not only reduce the uncertainty of hazard predictions, but can also lead to a causal understanding on how the assay results relate to a human adverse outcome and in which an adverse outcome pathway (AOP) is activated by the compound.

The NeuriTox assay, also called UKN4, was developed about 10 years ago, as a high-throughput NAM to assess developmental neurotoxicity (DNT) hazard [1,2]. It has since been used in a large European test battery [3], in a DNT screen of the U.S. National Toxicology Program (NTP) [4] and as part of the European Food Safety Agency (EFSA) test battery [5]. NeuriTox assesses acute neurotoxicity and DNT by quantifying the impairment of neurite outgrowth in differentiating dopaminergic neurons. The cells (LUHMES) used in the test procedure are neuronal precursors, originally derived from fetal brains [6,7]. For the assay, proliferating LUHMES cells can be further differentiated. They arrest their cell cycle, express biochemical markers of dopaminergic neurons, and develop a neurite network [8]. During this process, the maturing neurons switch their main pathway for energy metabolism from glycolysis to oxidative phosphorylation [9]. The NeuriTox assay endpoint is based on high-content imaging. This allows for the viability to be measured in parallel to neurite outgrowth. Data showing unspecific neurite degeneration as a consequence of cell death are automatically excluded [2]. In a blinded screen performed with the NTP library, seven compounds impaired neurite outgrowth without reducing cell viability: 1-methyl-4-phenylpyridinium (MPP), rotenone (Rot), colchicine (Col), diethylstilbestrol (DES), valinomycin (Val), berberine (Ber) and carbaryl (Car) [4]. A secondary screen was performed in iPSC-derived peripheral neurons, which essentially confirmed the NeuriTox hits [4], but did not yield additional mechanistic information.

Besides screening, the NeuriTox assay may be used for mechanistic studies if additional endpoints are introduced. For instance, mitochondrial or proteasome inhibitors have been characterized [10,11,12]. Such data have contributed to the development and testing of the adverse outcome pathway (AOP) on complex I (c-I) inhibitors leading to parkinsonian motor deficits (AOP:3) [12,13,14].

Complex phenotypic assays, like the NeuriTox, can capture many different modes of action. This has the advantage that compounds with different modes of action may be identified in screens. However, the downside is that the relevance of the hits resulting from the NeuriTox screen cannot be assessed with the original test prediction model. In order to better characterize the role of screen hits as potential toxicants, it is necessary to use additional test endpoints that yield information on modified signaling and cell regulation pathways. The experience on how such follow-up characterizations should be optimally organized is still very limited. One example for such an approach is the characterization of screen hits in a cell migration assay [15,16]. It was found that planar polychlorinated biphenyls (PCBs) impaired neural crest function by affecting cellular gap junctions. On this basis, it was possible to predict that other molecules with such a mode of action are likely to be toxic to neural crest cell migration [17]. Another example is the use of transcriptome profiling followed by signaling assays. This strategy helped to determine that compounds affecting JAK-STAT signaling (e.g., IFNβ) are likely to be neural crest toxicants [18,19].

The dearth of screen hit characterization studies is astonishing, given that mechanistic characterization of toxicants has a long tradition in toxicology. This field was well-established long before NAM became broadly available. The knowledge and experience of mechanistic toxicology needs to be leveraged to current screening approaches. Modern technologies are required for follow-up strategies linking screen hits to the AOP. The profiling of cell signaling, metabolism and transcriptome changes are important tools for hit characterization studies [20,21,22,23]. One example of the power of new methods is the advances in the characterization of energy metabolism. This has, e.g., allowed a large screen for mito-toxicants, followed by a mechanistic characterization of the hits as, e.g., uncouplers or c-I inhibitors [24]. Another example is the use of ToxCast data together with hit grouping to derive mechanistic information [25].

We used here the hits obtained from the NeuriTox NTP screen for an exemplary mechanistic study. Several follow-up technologies were combined to better understand cellular changes triggered by the compounds. The first goal of the study was to explore the complementarity of the layers of information derived from various approaches. The library screened for the US NTP contained (in blinded form) some well-known toxicants, in addition to data-poor compounds. For instance, Rot (mito-toxicant) and Col (microtubule disruptor) were included for their well-known toxicities. For such compounds, information spanning from molecular target interaction to in vivo adverse outcomes is available. As they were among the hits, they served as positive controls in the present mechanistic study. Our second goal was to unravel potential mechanistic commonalities of all seven hits, using unbiased approaches. In the second part of the study, we focused particularly on a subgroup of the screen hits: Rot, MPP and Ber. These three compounds were found to affect mitochondrial respiratory chain complex I (c-I). This confirmed the literature knowledge on positive controls Rot and MPP [26,27]. It also provided a basis for making Ber an interesting and relevant learning case: this compound emerged here from a phenotypic screen, and our follow-up data pointed to its mode of action. The literature data published during our mechanistic characterization study provided an additional layer of confirmation and showed that our approach can yield reliable data. In the last part of the study, we made use of the fact that three hits had a similar mode of action (c-I inhibition), and that we had gathered a multi-dimensional data set on these mechanistically related compounds. This offered the unique opportunity to define cellular changes specifically triggered by such mito-toxicants. Combined metabolomics-transcriptomics data were collected to highlight processes common to this group of compounds, and that may explain their neurite-specific hazard. The last part of this manuscript was assembled to compile information on specific events triggered by c-I inhibitors in dopaminergic neurons. Such information is important for the relevance and specificity of parkinsonian motor deficits triggered by c-I inhibitors (AOP:3). Our data set suggests new biomarkers and future endpoints of novel AOP-aligned NAMs.

## 2. Materials and Methods

### 2.1. Materials

Unless stated otherwise, cell culture consumables were acquired from Gibco/Thermo Fisher Scientific (Waltham, MA, USA).

### 2.2. LUHMES Cell Culture

The LUHMES are dopaminergic neuron precursor cells derived from a female human ventral mesencephalon and conditionally immortalized by v-myc. They were identified by STR typing and shown to have an intact chromosomal structure and ploidy by whole genome sequencing [7]. Upon differentiation, they form a dopaminergic neuronal culture, characterized by pronounced DAT activity, an up-regulation of tyrosine hydroxylase (TH) and exit from the cell cycle [8,9]. Culture and differentiation followed established protocols [5,7]. Briefly, proliferating LUHMES cells were maintained in PM medium (i.e., Adv. DMEM/F12 supplemented with 2 mM L-glutamine, 1 × N2 supplement (Invitrogen/Thermo Fisher Scientific, Waltham, MA, USA) and 40 ng/mL recombinant human basic fibroblast growth factor (Bio-Techne, Minneapolis, MN, USA)). Cells were kept in a humidified 5% CO_2_/95% air incubator at 37 °C and passaged every second day by trypsinization using 0.05% trypsin/EDTA (Invitrogen/Thermo Fisher Scientific, Waltham, MA, USA). To start differentiation, the medium was changed to DM (Adv. DMEM/F12 supplemented with 2 mM L-glutamine (Gibco, Rockville, MD, USA), 1 × N2 supplement (Invitrogen/Thermo Fisher Scientific, Waltham, MA, USA), 1 mM N6,2′-O-dibutyryl 3′,5′-cyclic adenosine monophosphate (cAMP), 1 µg/mL tetracycline (Sigma-Aldrich/Merck, Darmstadt, Germany) and 2 ng/mL recombinant human glial cell-derived neurotrophic factor (GDNF, Bio-Techne, Minneapolis, MN, USA). Cell culture plates and flasks (Sarstedt, Nümbrecht, Germany) were pre-coated with 1 μg/mL fibronectin and 50 μg/mL poly-L-ornithine (PLO) (Sigma-Aldrich).

### 2.3. LUHMES Differentiation and NeuriTox Exposure Scheme

The differentiation of LUHMES cells was performed as previously described [1,2,4]. For the transcriptomics experiment and ATP analysis, cells differentiated for 48 h (day 2, d2) were re-seeded into 96-well plates at a density of 200,000 cells/cm^2^ in DM. After 1 h, the treatment compound was applied to the cells, with exposure times ranging between 0 and 24 h. All the samples were collected simultaneously at the end of the experiment.

For the metabolomics experiment, for amino acid profiling and for Western blotting, the d2 re-seeding was conducted in 10 cm dishes at a density of 200,000 cells/cm^2^. Cells in these were treated in the NeuriTox assay with the test compounds for 24 h. The control samples were treated with 0.1% dimethyl sulfoxide (DMSO, Merck, Darmstadt, Germany) for 24 h.

### 2.4. Chemical Compound Descriptions

To facilitate the interpretation of our findings, we provide detailed descriptions of the selected toxicants in Supplementary Figure S1. The selected toxicants and their respective mechanisms of action are summarized below.

Rot and MPP are well-known mitochondrial toxicants (c-I inhibitors). Col is a microtubule disruptor that has often been used as positive control for the NeuriTox test [4]. Car is best known for inhibition of acetylcholine esterase, but it may also have other intracellular targets. DES is a potent agonist of the estrogen receptor, but it has also non-estrogenic activities when used at high concentrations. For Val, the primary mode of action is to permeabilize membranes to K^+^. Cellular consequences could range from mitochondrial uncoupling to necrosis. Ber was, on the one hand, described as a mitochondrial inhibitor, but on the other hand, it is considered to be safe for human use. This hit therefore appeared particularly interesting for further characterization of its MoA, and for comparison with the other compounds.

All test compounds were acquired from Sigma-Aldrich/Merck (Darmstadt, Germany).

### 2.5. Sample Preparation for the Metabolomics Experiment

The processing was described in detail before [11]. Briefly, cells were lysed in 80% methanol using cell scrapers. The residual cell lysate was subsequently collected in a second scraping step in 80% methanol. Lysates were stored at −80 °C and thawed. Then, samples were homogenized and centrifuged (30 min, 20,000× *g*, 4 °C). The supernatant was placed in a Savant SpeedVac concentrator (Thermo Fisher Scientific, Waltham, MA, USA) for drying, whereas the methanol-precipitated pellet was used for the measurement of protein content.

### 2.6. Liquid Chromatography–Mass Spectrometry (LC-MS) Analysis

Samples were shipped on dry ice for analysis by Metabolon (Research Triangle Park, NC, USA). A non-targeted approach was employed to generate the metabolic profiles utilizing UPLC (ultra-performance liquid chromatography) coupled with MS/MS (tandem mass spectrometry). In order to detect a multitude of metabolites with wide-ranging physico-chemical properties, four different methods were used in parallel: (1) reverse phase (RP)/UPLC-MS/MS under positive ionization, (2) RP/UPLC-MS/MS under positive ionization optimized for more hydrophobic compounds, (3) RP/UPLC-MS/MS under negative ionization and (4) HILIC/UPLC-MS/MS (negative ionization). All analyses were conducted using a Waters ACQUITY ultra-performance liquid chromatography (UPLC) and a Q-Exactive high resolution/accurate mass spectrometer interfaced with a heated electrospray ionization (HESI-II) source and Orbitrap mass analyzer operated at 35,000 mass resolution (Thermo Fisher Scientific, Waltham, MA, USA). The instrument used dynamic exclusion to alternate between MS and MS/MS scans and covered a range of 70–1000 *m*/*z*.

Data extraction, peak identification and quality controls were performed by Metabolon using its own hardware and software. The identification of metabolites was achieved by the laboratory information management system (LIMS), which compared the paired retention time/index (RI)–mass-to-charge ratio (*m*/*z*) against a library of more than 3300 purified standards.

Data analysis and visualization were conducted using the R software version 4.1.1. For each sample, the metabolite data were normalized to the average of the DMSO controls. The statistical analysis was conducted using a moderated *t*-test statistics implemented by the R package limma [28]. The Benjamini–Hochberg method was applied to correct for multiple testing. Metabolites with an adjusted *p* value < 0.05 were considered significant. The complete data matrix can be found in Supplementary Table S1.

### 2.7. ATP Measurement

Intracellular ATP was determined as described earlier [9,29]. Briefly, cells were lysed by adding to the medium the luciferase-containing CellTiterGlo 2.0 mix (Promega, Madison, WI, USA). The data obtained from measuring luminescence were background-corrected, then normalized to DMSO controls.

### 2.8. Image Analysis of Viable Cells

Image acquisition of LUHMES cells live-stained with 1 µg/mL Hoechst H-33342 (Sigma Aldrich, Steinheim, Germany) and 1 µM calcein-AM (Biomol GmbH, Hamburg, Germany) was conducted by an automated high-content imager (Cellomics VTI, Waltham, MA, USA), as described previously [1,2,30].

### 2.9. Oxygen Consumption Rate and Extracellular Acidification Rate

The procedure was used as detailed earlier [11,29]. Briefly, LUHMES cells were plated on d2 in PLO/fibronectin-coated “Seahorse 24-well plates” (Agilent, Santa Clara, CA, USA) at a density of about 100,000 cells/well. After 1 h, they were treated with the test compound for 24 h. Then, medium was replaced with Agilent Seahorse XF DMEM medium, pH 7.4, supplemented with 18 mM glucose, 2 mM glutamine and 1 mM pyruvate. One hour later, basal mitochondrial oxygen consumption was first assessed using the Seahorse XFe24 analyzer from Agilent (Santa Clara, CA, USA). Finally, the Mito stress test was performed, by taking several measurements after injecting each with (i) 1 μM oligomycin, (ii) 1.5 μM carbonyl cyanide-4-(trifluoromethoxy)phenylhydrazone (FCCP) and (iii) 0.5 μM rotenone with 0.5 μM antimycin A. For each well, the exact number of cells was quantified and used to normalize the oxygen consumption rate. Individual mitochondrial parameters were determined after normalization according to the manufacturer’s instructions.

### 2.10. Protein Determination

The total protein concentration was determined using a BCA protein assay kit (Pierce/Thermo Fisher Scientific, Rockford, IL, USA). The pellets obtained by methanol precipitation were re-suspended in 1 mL 100 mM NaOH and further incubated on a shaker at 50 °C overnight. For the BCA assay, samples were first diluted 1:10.

### 2.11. Quantification of Signaling Proteins

For sample preparation, LUHMES cells were lysed in Laemmli buffer and boiled at 95 °C for 5 min. The lysate was centrifuged for 1 min at 10,000× *g* through NucleoSpin Filters (Macherey-Nagel, Düren, Germany) to remove nucleic acids. The DigiWest method was used exactly as described earlier [31]. Briefly, the analysis procedure started with an electrophoretic separation and blotting of proteins onto nitrocellulose membranes. Then, proteins were biotinylated and eluted from small pieces of the membrane (each lane was cut into 96 evenly large strips). Eluted proteins were incubated with color-coded streptavidin-coated Luminex beads (Luminex FlexMAP 3D system). Beads were incubated with primary antibodies for the selected proteins (acetylated tubulin; phosphorylated (at serine-51) and non-phosphorylated eIF2-alpha) and a phycoerythrin-labelled secondary antibody (all reagents and devices by the Thermo Fisher LUMINEX platform (Waltham, MA, USA)).

### 2.12. In Vitro Tubulin Polymerization Assays

The tubulin polymerization rate was determined by the change in absorbance at 340 nm over time [32]. Purified pig brain tubulin was diluted in a glutamate buffer (0.8 M, pH 6.6) to 10 μM and supplemented with MgCl2 (100 μM) and DMSO or compounds (with indicated concentrations) on ice. After pre-incubation at 30 °C, reactions were put back on ice for 10 min, and GTP (0.4 mM) was added to the mixture. Polymerization was initiated by raising the temperature to 30 °C, and absorbance (340 nm) was measured every 20 s over 30 min (TECAN Infinite F500 multimode reader).

### 2.13. Amino Acid Analysis

The amino acid analysis was conducted as described previously [9]. Washing of the 10 cm cell culture dishes was performed once with PBS, followed by quenching with 50% *v*/*v* methanol/H2O. The resulting solution was incubated at 4 °C in an Eppendorf Thermomix (Hamburg, Germany) at 1400 rpm for 30 min, then centrifuged for 15 min at 21,000× *g* at 4 °C. The supernatant was transferred to new tubes and dried in the SpeedVac concentrator. Samples were re-suspended in 2% (*w*/*v*) 5-sulfosalicylic acid (SSA), then centrifuged for 20 min at 1440 rpm, 4 °C. The supernatant was centrifuged for 10 min at 20,000× *g*, 4 °C. Utilizing the Sykam S433 amino acid analyzer (Sykam, Fürstenfeldbruck, Germany), the separation of amino acids was carried out by HPLC (lithium-based anion exchange column: 7 µm diameter, 10% cross-links, cat# 5125022) and post-column derivatization with ninhydrin. Elution was performed using buffers with increasing pH (pH 2.9→pH 12), ion strength (buffer concentration 0.12–0.45 M) and using a temperature gradient. The reaction products were quantified at 570 nm (most amino acids) and at 440 nm (for the intermediate products in the case of cysteine and proline). The area under the peak was quantified using the ChromStar version 7.0 software (SCPA, Weyhe-Leehste, Germany) and by comparison to reference standards, the concentration was determined.

### 2.14. Determination of Glucose and Lactate in Cell Culture Medium

The quantification of glucose and lactate was performed using a colorimetric cuvette assay (GLU-142 and LAC-142, respectively; Diaglobal, Berlin, Germany). Briefly, enzymatic conversion of glucose or lactate resulted in the formation of colorimetric products, which were subsequently quantified through spectrophotometric measurements and compared to calibration standards.

### 2.15. Sample Preparation for the Transcriptomics Experiment

For the time profiling, the NeuriTox EC10_V_ concentrations were used: 64 µM carbaryl (Car), 40 nM colchicine (Col), 94 µM berberine chloride (Ber), 21 µM diethylstillbestrol (DES), 4.4 µM rotenone (Rot), 10 µM MPP^+^ and 17 nM valinomycin (Val). For sample preparation, the medium in 96-well plates was replaced by Biospyder Lysis Buffer (33 µL/well; Biospyder Tech., Glasgow, UK). Following a 15 min incubation at room temperature, the plates were sealed and frozen at −80 °C for lysis completion.

Targeted transcriptome sequencing (including QC, alignment and read quantification) was conducted at Bioclavis (Biospyder Tech., Glasgow, UK) using the TempO-Seq technology in combination with the EU-ToxRisk v2.1 probe panel [33]. For each of the 3257 targeted genes, a 50 bp fragment was amplified, while also introducing sample-specific barcodes, which subsequently enabled sample pooling for the next-generation sequencing of the collection. A reference library containing the collection of all amplification products was used for assigning read counts to each targeted gene. A pre-filtering step for library size (<0.2 million) and average gene count (<1.5) was performed. Replicates from the highest tested concentration of DES (42 µM) were excluded during the filtering step due to their low library size, potentially indicating cytotoxic effects. The counts per gene were normalized to counts per million by dividing by the total number of mapped reads per sample and multiplying by 10^6^. The effect of normalization was checked by boxplots and distribution plots (not shown) and no outlier samples were identified. The differential gene expression (DGE) analysis of each treatment against the control group was conducted by the Wald test implemented in DESeq2/R [34], including a FDR correction using the Benjamini–Hochberg algorithm. Significant transcriptomic changes were identified by differential gene expression analysis: each compound exposure time and concentration was contrasted against the control. Each time point had its own DMSO control.

The complete data matrix can be found in Supplementary Table S1. To check for functional enrichment, we applied the WMEAN algorithm from decoupleR [35] on the gene expression statistics (stat) mapped onto DoRothEA regulons [36].

### 2.16. Curve Fitting and Statistics

All omics samples analyzed in this study were biological replicates, and were thus treated as statistically independent. For amino acid analysis, three treated samples per time point were used; the untreated control values are based on four samples. For the metabolomics analysis, four treated samples per time point were used; the untreated control values are based on six samples. For the transcriptomics analysis, six samples were produced and measured for each condition. Data of treated samples are expressed relative to solvent (DMSO) controls. If not mentioned otherwise, data displayed are the means ± SEM; in addition, individual data points are displayed where this makes the data structure more transparent. The plots were created using GraphPad Prism 7.0 (GraphPad Software, La Jolla, CA, USA). Correlated activity scores were calculated on the Wald statistic (stat) using the weighted mean for all experimental conditions. Where other statistics were used, this is indicated in figure legends. Significance levels are indicated by asterisks: * *p* < 0.05, ** *p* < 0.01, *** *p* < 0.001. When EC25(viability) was > the highest tested concentration (HTC), then 2 × HTC was used as surrogate EC25 for some ratio calculations.

## 3. Results and Discussion

### 3.1. Experimental Design to Follow Up on a Toxicity Screen Based on Maturing Dopaminergic Neurons

**Figure 1:**
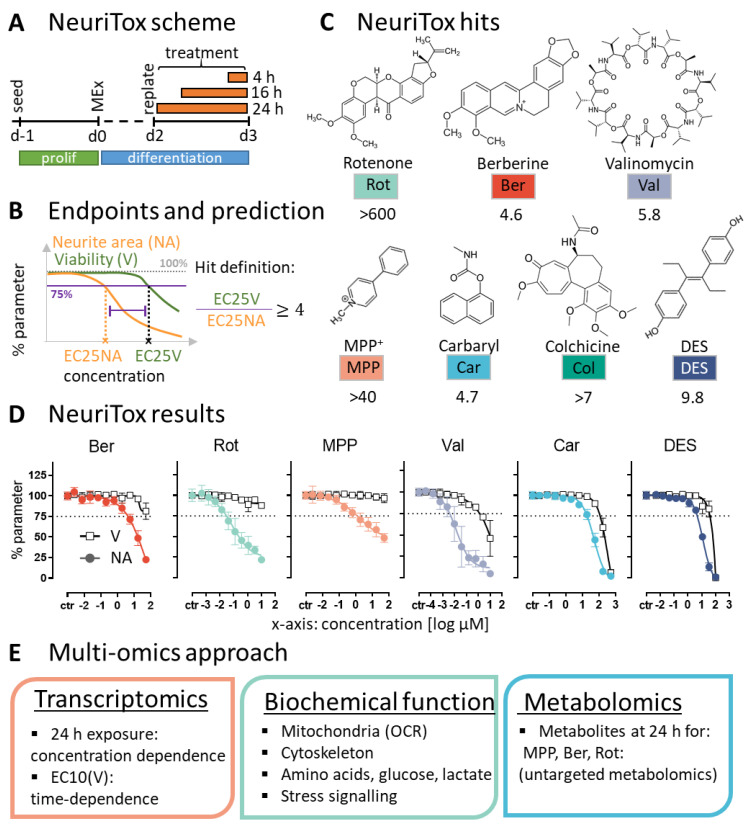
Overview of the study design and the compounds investigated. The seven compounds studied here were originally identified in an earlier screen, using the NeuriTox assay [4]. For follow-up studies, differentiating LUHMES neurons were exposed on day 2 (d2) to the screen hits for up to 24 h. (**A**) General assay scheme: pre-differentiated LUHMES cells were seeded on d2 and treated 1 h later with the toxicants. Orange bars indicate the exposure duration. For determining the toxicity threshold (benchmark concentrations), the 24 h exposure was performed using serial dilutions of the test compounds. (**B**) The overall viability- (V) and neurite area (NA) inhibition were quantified, using the NeuriTox/UKN4 assay, and the concentrations leading to a NA reduction by 25% (EC25_NA_) were determined. Hits are defined as compounds which decreased viability by 25% (EC25_V_) at a concentration at least 4 x higher than the EC25_NA_. (**C**) The seven previously identified hits were confirmed here as specific neurite outgrowth inhibitors in an assay run with smaller concentration steps than in the screen mode. The V/NA ratios are given for: rotenone (Rot), berberine chloride (Ber), valinomycin (Val), 1-methyl-4-phenylpyridinium (MPP^+^), carbaryl (Car), colchicine (Col), diethylstilbestrol (DES). The EC25_V_/EC25_NA_ is indicated below each compound. (**D**) Graphs show concentration-dependent changes in neurite area (NA—colored circles) and viability (V—empty squares). The dotted line indicates a benchmark response of 25%. The EC25_NA_ concentrations are as follows: 4 µM Ber, 30 nM Rot, 1 µM MPP, 4 nM Val, 19 µM Car and 5 µM DES. Data are means ± SEM of averaged data from independent experiments (n = 3; N = 3). (**E**) Schematic illustration of the three main approaches used for further investigation of the compounds’ mode of action: (i) transcriptomics profiling in a concentration- and time-dependent manner, (ii) assessment of effects on biochemical cell functions and (iii) metabolome profiling after 24 h exposure to each of the complex I inhibitors (MPP, Ber, Rot). Biochemical functions investigated included: mitochondrial oxygen consumption rate (OCR) and changes in the abundance of intracellular amino acids.

To characterize the mode of action (MoA) of the hits from an environmental toxicants screen, we designed a multi-omics approach. LUHMES cells were treated essentially similar as in the screen: toxicants were added on day 2 of differentiation (d2) for up to 24 h (Figure 1A). At this stage, the neuronal precursor cells are in the process of acquiring the neuronal dopaminergic phenotype. For instance, they upregulate the dopamine transporter (DAT) and dopamine receptors (DRD2, DRD4) [8,37]. On d3 cells display an established neurite network (positive for beta-III tubulin) and they express vesicular monoamine transporter-2 (VMAT) [1].

The NeuriTox readout uses high-content imaging to quantify changes in viability (V) and neurite area (NA). For a test compound to be classified as DNT-specific, the neurite area benchmark concentration (EC25_NA_) has to be at least four times lower than for viability (BMC25_V_) (Figure 1B). Seven compounds of the NTP library met this criterion [4]. In the current study, the hits were re-analyzed, and robust concentration-effect data confirmed the screen results (Figure 1C,D). The compounds are diverse and data-rich (Supplementary Figure S1). This was considered useful for a proof of concept study. The unbiased multi-omics approaches could, in this way, be controlled and partially guided by some basic knowledge on the chemical’s activities. To obtain rich data sets, we analyzed transcriptome changes, checked for alterations in the biochemical functions and checked metabolic perturbations of the neurons (Figure 1E).

### 3.2. Overall Patterns of Transcriptome Changes over Time

**Figure 2:**
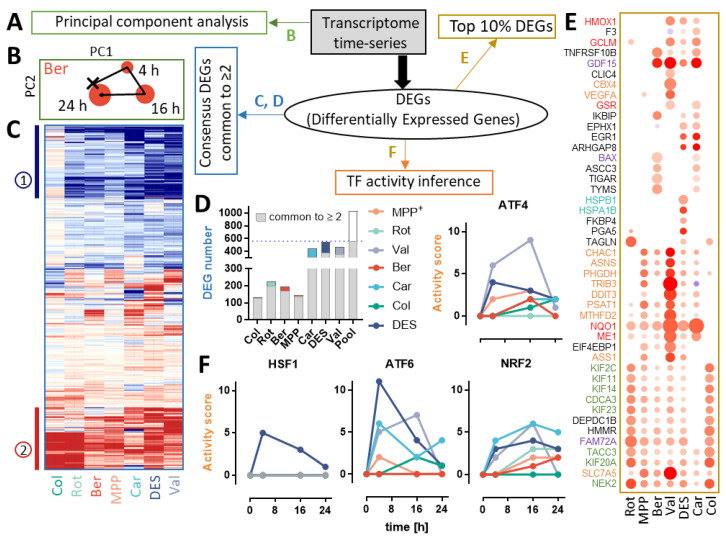
Time-dependent transcriptome changes induced by neurotoxicants. Transcriptome data were obtained from LUHMES cells treated with seven different neurotoxicants for 4 h, 16 h and 24 h, as outlined in Figure 1A. The following concentrations were used: 64 µM carbaryl (Car), 40 nM colchicine (Col), 94 µM berberine chloride (Ber), 21 µM diethylstilbestrol (DES), 4.4 µM rotenone (Rot), 10 µM MPP+ and 17 nM valinomycin (Val). As output metrics, we provide the average log2 fold changes (FCs) relative to the solvent control of the respective time point for each transcript, including the standard deviation, and the statistical significance of the change in Supplementary Table S1. (**A**) Graphical overview of the data analysis: transcriptome time-series data were used as input for principal component analysis (PCA) and to determine differentially expressed genes (DEGs). The latter were used for more refined downstream analyses, as indicated by subfigure letters. (**B**) Log2 FC data from all transcriptome conditions (3 times × 8 treatments) were analyzed together in a joint principal component space. The data for berberine (Ber) are shown here, while the complete PCA plot is displayed in Figure S2A. The “×” denotes untreated cells, the time labels indicate data points after respective exposure times to Ber. The circle diameters correspond to the exposure time. (**C**) For each compound and gene, the expression change was followed over time, and only the peak data (defined by significance) were kept as a dimensionality-reduced data set. The genes with an adjusted *p*-value ≤ 0.05 and an absolute FC > 1.5 were considered “consensus genes” and selected for further analysis (Figure S2B). Those shared by ≥ 2 compounds (561 out of 3255 measured genes) were selected for further analysis. The heat map gives an overview of the clustered gene hits (common to at least two treatments). The color scale spans from blue (four-fold down) over white (no regulation) to red (four-fold up). Two heat map regions (1, 2) were considered interesting for further analysis and more detailed overviews (shown in Figure S2C). (**D**) The bar graph shows the number of differentially expressed genes per compound. The proportion of consensus genes is marked in gray. The colored parts of the bars indicate genes specifically affected by the individual compounds. Pool: data on all genes regulated by at least one toxicant (n = 1037). (**E**) The top 10% up-regulated genes per compound were selected and displayed together in a dot plot, where significance is encoded by circle size. Red colors indicate up-regulation (full red = four-fold). Blue colors (very rare) indicate down-regulations. Genes were mapped to five functional categories, marked by different colors: cell cycle regulation and mitosis (green), mitochondrial stress response orchestrated by ATF4 (orange), oxidative stress response orchestrated by NRF2 (red), heat shock response (blue) and cell death/parkinsonism (purple). (**F**) The activity of transcription factors (TF) was predicted based on target gene expression [36]. Predicted activity scores of ATF4, HSF1, ATF6 and NRF2 were plotted over time. For ATF4, the individual regulation of its targets used for the prediction is shown for the 16 h time point in Figure S3. Note that detailed changes are extensively described in supplementary results 1.

Data on transcriptome changes were obtained for three exposure times. Already after 4 h, strong changes were evident, and they increased over time (16 h), but rather returned towards baseline after 24 h (Figure 2A,B). The similar time-tracks on a 2D PCA map indicate a large degree of similarity in the transcriptome responses of diverse compounds.

In a next step, we were interested in a comparison of toxicant responses on a gene-by-gene level. All genes that were changed by at least two compounds were selected for a display. Two clusters of similarly behaving genes emerged (Figure 2C): stress response markers (e.g., *NQO1*, *CHAC1*) and cell cycle regulators (cyclins *CCNB1*, *CCNB2*; kinases *PLK1* and *AURKA*; components of the spindle assembly checkpoint *BUB1* and *BUB1B*; kinesins *KIF20A*, *KIF23*, *KIF11*, *KIF15*) (Figure S2C). Car, DES and Val had the most pronounced effect on the transcriptome (Figure 2D and Figure S2D,E).

Stress-response genes were abundant amongst the top 10% genes up-regulated by each compound (Figure 2E,F). Expression of NRF2 targets (*NQO1*, *ME1*) was elevated by all treatments, except Col. Genes under the control of ATF4 (*CHAC1*, *ASNS* and *MTHFD2*) were induced by MPP, Ber, Val, DES and Car. DES uniquely enhanced the expression levels of HSF1-regulated genes (*HSPB1* and *HSPA1B*). At 16 h, the activity of ATF4 and NRF2 was predicted to be significantly increased by most toxicants (Figure 2F, Figures S3 and S4). Analysis of overrepresented pathways indicated a change in transsulfuration (Figure S2E), which is a typical outcome of ATF4/NRF2 activation and confirms a general stress response.

### 3.3. Concentration-Dependent Transcriptome Changes Induced by DNT Compounds

**Figure 3:**
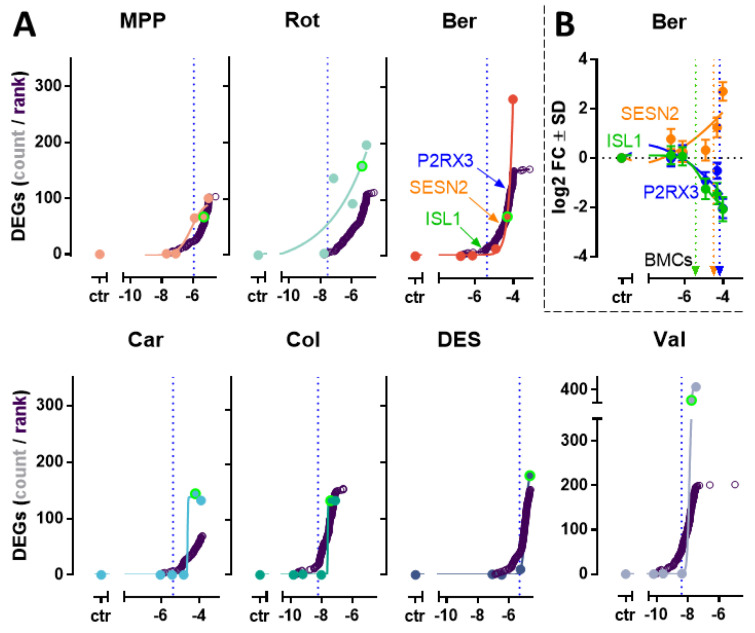
Concentration-dependent transcriptome changes induced by exposure of neurons to toxicants. Transcriptome data were obtained from LUHMES cells treated for 24 h with seven different toxicants at five different concentrations. The five concentration intervals were anchored to cytotoxicity data. Concentrations corresponded to 2 × EC_10_, EC_10_, 0.25 × EC_10_, 0.015 × EC10, 0.004 × EC10. The following concentrations intervals were used: 0.25–130 µM carbaryl (Car), 0.15–77 nM colchicine (Col), 0.18–94 µM berberine chloride (Ber), 0.08–42 µM diethylstilbestrol (DES), 0.02–8.7 µM rotenone (Rot), 0.02–10 µM MPP+ (MPP) and 0.07–33 nM valinomycin (Val). The vertical dotted line indicates the benchmark concentration corresponding to a 25% reduction in neurite area (EC25_NA_) as derived from Figure 1. Differentially expressed genes (DEGs) had to meet the following criteria: adjusted *p*-value < 0.05 and absolute FC > 1.5. All background data (average log2 fold changes (FC) relative to the solvent control of the respective compound concentration for each transcript, including the standard deviation, and the statistical significance of the change) are found in Supplementary Table S1. (**A**) The filled circles indicate the number of DEGs. For clarity, the data point of the EC10_V_ is shown with an extra green circle. As alternative approach, benchmark concentrations (BMC) were calculated in BMDExpress for all genes with a monotonic expression over the concentration series. The genes were ranked by potency and assigned a rank number. Then, the gene BMCs were plotted against rank orders to yield the function of the accumulation plot (empty circles, purple). The *x*-axis represents the concentration on the log molar scale. For Ber, the identity of three exemplary genes of the accumulation plot is indicated by the arrows (**B**) Concentration-response curves for the 3 exemplary genes shown in A. The *y*-axis represents fold changes (FC) on the log2 scale. Dotted vertical lines mark the BMC of the corresponding gene (indicated by color code). Data are means ± SD of independent experiments (N = 3). The *x*-axis represents concentrations on the log M scale.

We tested a range of concentrations to characterize the transcriptome changes at 24 h (the exposure time used in the NeuriTox assay) (Figure 3A). The response (in number of DEGs) was concentration-dependent for all test compounds. Notably, gene expression was hardly affected at non-cytotoxic concentrations; only MPP and Rot showed a small response. As a standardized approach to display all changes on the level of individual genes, we determined the benchmark concentration (=BMC_gene-x_) for each transcript (Figure 3B). The “accumulation plots” of the ranked BMC_gene-x_ closely resembled the profile of the DEG curves (Figure 3A). The accumulation plot display was adopted, from [38], as this has become a standard in the display of toxicogenomic responses [39]. When we compared the transcriptome-response to the BMC25_NA_, we observed that the 24 h neurite outgrowth endpoint is more sensitive than the gene regulation endpoints. Concerning the specificity and sensitivity of the toxicogenomics approach, our findings suggest that: (i) gene regulation is not an over-sensitive (low specificity) endpoint, when applied to the NeuriTox test system, and (ii) the onset of neurite toxicity correlated well with the onset of some transcriptome response. Strictly speaking, these findings refer to the 24 h time point. It is possible that low test compound concentrations (≤BMC25_NA_) triggered a transient response between 4 and 16 h.

### 3.4. Changes in Glycolytic Rate and Mitochondrial Respiration

**Figure 4:**
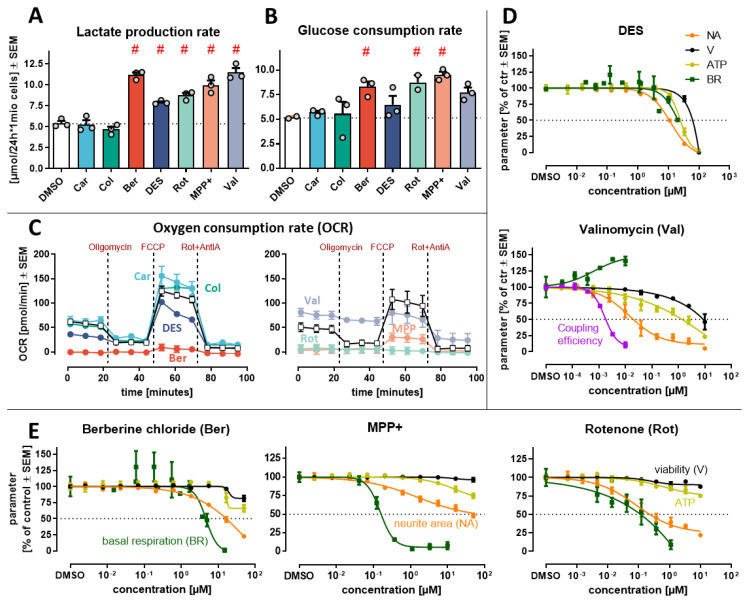
Effect of seven toxicants on glycolysis and mitochondrial respiration. Immature neurons (day 2 LUHMES) were exposed for 24 h to developmental neurotoxicants (DNT): 20 µM carbaryl (Car), 100 nM colchicine (Col), 15 µM berberine chloride (Ber), 10 µM diethylsylbestrol (DES), 1 µM rotenone (Rot), 10 µM MPP^+^ and 10 nM valinomycin (Val). (**A**) Bar plots show the neuronal lactate production and (**B**) the glucose consumption rates, as determined from medium measurements. The ratios between these two parameters are displayed in Figure S7. Independent replicates are represented as dots. Data are means of three independent replicates ± SD. Statistical differences between the treatments and the control were evaluated by an analysis of variance (ANOVA) followed by Dunnett’s post hoc test (*p* < 0.05, indicated by #). (**C**) Effect on mitochondrial respiration was investigated through the “Mito stress” test at the indicated concentrations. (**D**,**E**) The compounds showing activity on mitochondrial respiration were further tested in concentration series up to the highest non-cytotoxic concentration (EC10V), from which the basal respiration parameter was derived. Intracellular ATP was measured for the five OCR-modulating compounds and plotted together with viability (V), neurite area (NA), basal respiration (BR) as concentration–response curves. For Val, the coupling efficiency is also displayed. The ATP measurements for the 2 non-mitochondrial toxicants are shown in Figure S7. Data are means ± SEM of independent replicates. AntiA—antimycin A; DES—diethylstilbestrol; FCCP—carbonyl cyanide-p-trifluoromethoxy-phenylhydrazone; MPP^+^—1-methyl-4-phenylpyridinium.

Mitochondrial dysfunction, primary or secondary, is a common feature of neurodegeneration and neurotoxicity. A co-activation of NRF2 and ATF4 stress responses, as observed here, is one of the indicators of mito-toxicity [40]. We therefore assessed mitochondrial dysfunction, and also glycolysis, as a major compensatory pathway. As a proxy for glycolytic rate, we measured changes in the extracellular levels of the main substrate glucose. We also assessed lactate production, a metabolic pathway closely associated with mitochondrial dysfunction. Ber, MPP and Rot increased the glucose consumption rate. At the same time, lactate levels in the medium increased (Figure 4A,B). The rate of glucose consumption was similar to the rate of lactate production, which suggests that they are together indicative of an increased glycolytic flow (Figure S7A). This is the typical compensatory response, when mitochondria fail. DES and Val increased only lactate production, without a significant increase in glucose consumption. Such a different pattern of metabolic re-routing suggests other main targets of these toxicants.

As there was initial evidence for an altered energy metabolism for several toxicants, we checked directly for effects on mitochondrial respiration. The oxygen consumption rate (OCR) was measured after 24 h exposure to the toxicants. Rot and MPP were confirmed as strong inhibitors. In addition, Ber showed a pronounced effect (virtually complete block of basal respiration) (Figure 4C). DES also reduced basal respiration, however less potently (Figure 4D). Val increased the basal respiration by 30% at a concentration of ~3 nM (Figure 4C,D). Such a response indicates mitochondrial uncoupling. Car and Col clearly did not affect mitochondrial respiration (Figure 4A–C and Figure S7A,B).

For the three compounds which strongly inhibited mitochondrial respiration (Ber, MPP, Rot), we assessed how this affected the cellular ATP levels (Figure 4E). For all three compounds, we identified concentrations at which respiration was inhibited, but ATP did not drop. Our data suggest that the increased glycolytic rate (observed in Figure 3A,B) successfully buffered ATP production, even at compound concentrations which completely abolished mitochondrial respiration.

Overall, this analysis indicated that toxicants may trigger similar and general stress response pathways, although they differ in their targets. For instance, cellular oxidative stress may have mitochondrial or non-mitochondrial sources. Also, ATF4 may be triggered via PERK by ER stress or via HRI by mitochondrial stress [41].

### 3.5. In Vitro Inhibition of Tubulin Polymerization

**Figure 5:**
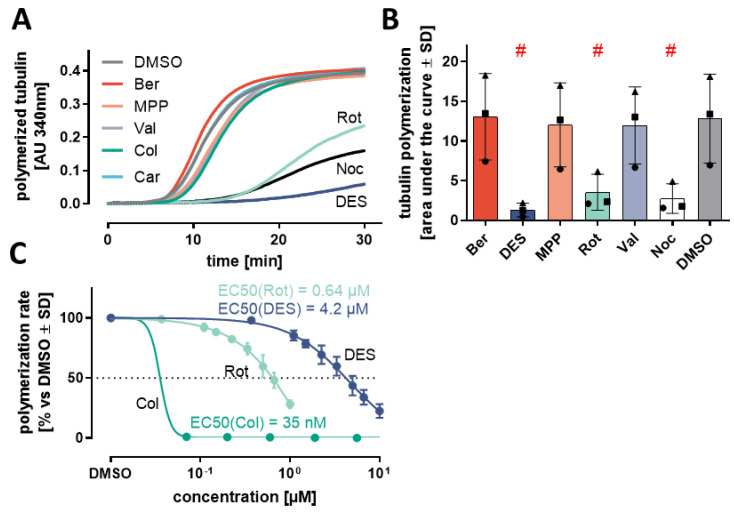
Effects of toxicants on tubulin polymerization. A biochemical assay based on purified tubulin protein monomers was used to study tubulin polymerization in the absence or presence of toxicants. (**A**) The polymerization assay was performed at 30 °C in the presence of the test compounds (20 µM carbaryl (Car), 100 nM colchicine (Col), 15 µM berberine chloride (Ber), 10 µM diethylsylbestrol (DES), 1 µM rotenone (Rot), 10 µM MPP^+^ and 10 nM valinomycin (Val)). The reaction was monitored continuously in a spectrophotometer at 340nm (arbitrary units, AU) for 30 min. Nocodazole (Noc, 1 µM) served as a positive control. The negative control treatment contained 0.5% DMSO and was used for background correction. Exemplary curves are shown. (**B**) The area under the curve (AUC) was calculated for three independent replicates and displayed as bar plots. (**C**) The concentration-dependent inhibition of microtubule polymerization was assessed for Rot, DES and Col at various concentrations. Data were normalized to the DMSO control, and the concentration leading to a half-maximal inhibition (EC50) was determined from curve fits. Data are means of three independent replicates ± SD. Statistical differences between the treatments and the control were evaluated by ANOVA followed by Dunnett’s post hoc test (*p* < 0.05, indicated by #). More data are found in Figure S8. FC—fold change.

Many previous hits in neurite elongation assays interfere with cytoskeleton dynamics [10,42]. Microtubules are particularly important for neurite functioning [43] because of their role in axonal transport [44]. Moreover, cytoskeleton-related proteins (e.g., kinesins) ranked high among the up-regulated genes identified here. Therefore, we checked our screen hits for interference with the microtubule dynamics. Rot, DES and Col inhibited microtubule assembly (Figure 5A–C and Figure S8A,B). This was expected for Col, an archetypical spindle poison. The findings on Rot and DES may appear surprising, but they confirm some earlier reports that described microtubules as additional targets relevant for the toxicity of these compounds [45,46].

To study an effect of toxicants on microtubule dynamics within the cell model used here, we measured tubulin acetylation. Altered levels and localizations of this post-translational modification are found in many neurodegenerative diseases [47]. Indeed, the same three compounds that affected microtubule dynamics in the biochemical assay also elicited significant changes in tubulin acetylation levels in live cells (Figure S8C). The amount of acetylated tubulin increased in the presence of Rot and DES. Col had the opposite effect, reducing the amount of modified tubulin to <20%. This is in line with previous observations and it also shows that the toxicants may affect microtubules in different ways and via different mechanisms [48].

Altogether, the results from these initial profiling studies suggest that the hits of our phenotypic screen (neurite growth) affect cells differently at a molecular level. Within the toxicants studied here, mitochondrial inhibitors formed an obvious subgroup. We hypothesize that compounds triggering similar cellular and metabolic effects would share a toxicological target pathway. This means that our data would suggest Ber (similarity to Rot and MPP^+^) to be a c-I inhibitor, even in the absence of any data in the literature. We explored how such a similarity-based target prediction may be refined.

### 3.6. Changes in Amino acid Metabolism

**Figure 6:**
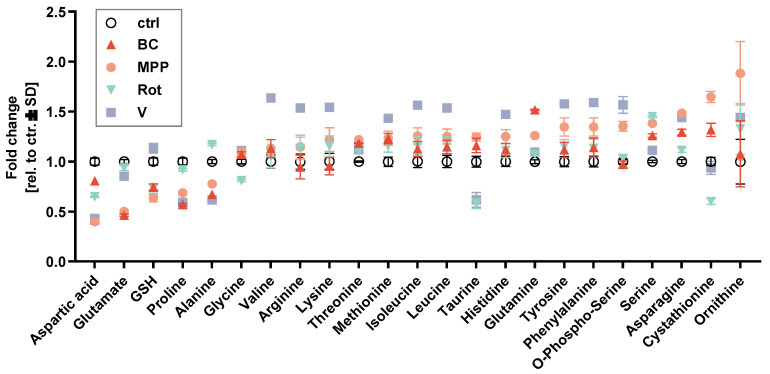
Intracellular amino acid changes in immature neurons exposed to neurodevelopmental toxicants. LUHMES cells (d2) were exposed for 24 h to putative neurodevelopmental toxicants: 10 µM berberine chloride (Ber), 10 µM MPP^+^, 1 µM rotenone (Rot), 20 µM carbaryl (Car), 100 nM colchicine (Col), 10 µM diethylstilbestrol (DES) and 10 nM valinomycin (Val). Cell lysates were prepared and intracellular amino acid levels were measured by HPLC. Data were normalized to the solvent control (DMSO). Changes in intracellular amino acid levels are displayed as fold changes (FC) for selected toxicants (Ber, MPP^+^, Rot and Val). Data are means ± SD from at least three independent experiments.

We used HPLC-based post-column-derivation amino acid analysis [49] to check intra-cellular amino acid concentrations. The response patterns of mitochondrial toxicants (MPP, Ber, Rot and Val) were indeed largely similar (Figure 6). For instance, ornithine (Orn), asparagine (Asn), glutamine (Gln) and serine (Ser) were increased, while aspartate (Asp) and glutamate (Glu) were depleted. When we calculated the Asn/Asp ratio, we found that all toxicants (besides Car) increased it by at least 1.5-fold (Figure S9). The change was significant for all mitochondrial toxicants. This may indicate a shift in nitrogen metabolism.

### 3.7. Global Metabolic Changes Induced by Mitochondrial Toxicants

**Figure 7:**
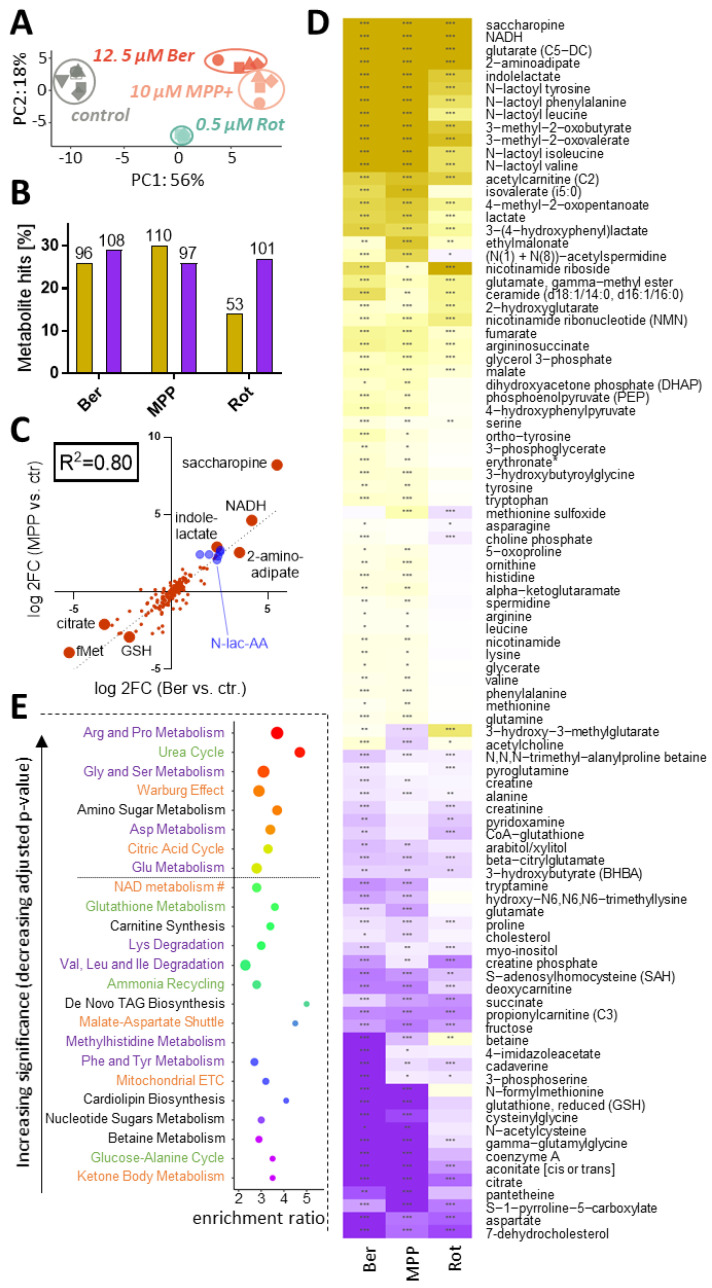
Overview of metabolic changes in immature neurons exposed to mitochondrial toxicants. LUHMES cells (d2) were exposed to 12.5 µM berberine (Ber), 10 µM MPP^+^ (MPP) or 0.5 µM rotenone (Rot) for 24 h. Intracellular metabolite concentrations were determined by LC-MS. Significant metabolite changes (vs solvent control) were identified for each treatment condition. (**A**) Principal component analysis (PCA) of the whole data set: the axes are scaled according to the variances covered. Each biological replicate is displayed with a different symbol. (**B**) Number of up-regulated (yellow) and down-regulated (blue) metabolites reported as % of all (369) detected metabolites (y-axis) and as absolute numbers (above bars). (**C**) The changes of metabolites triggered by Ber and MPP were related to one another in a scatter plot. All detected metabolites, besides lipids, dipeptides, NA-AA and nucleotides are shown (n = 180). (**D**) The heatmap displays all metabolites changed significantly by at least two toxicants (n = 85). Note that lipids, dipeptides, NA-AA and nucleotides are separately displayed in Figure S10. Yellow colors indicate up-regulation (saturated yellow = 4-fold), purple colors indicate down-regulations (saturated purple = 0.25-fold). Asterisks indicate the significance levels. Exact numbers are found in Supplementary Table S1. (**E**) Significantly changed metabolites (except lipids) were contrasted against the KEGG database, to find over-represented pathways. All pathways that are listed have *p*-values < 0.05 and are ordered by significance. After Benjamini–Hochberg correction for multiple testing, only the pathways above the dotted line had an adjusted *p*-value < 0.05. Pathways were grouped into four categories: classical amino acid (AA) metabolism (purple), AA-related metabolism (green), energy metabolism (orange) and other (black). * adjusted *p*-value ≤ 0.05; ** adjusted *p*-value ≤ 0.01; *** adjusted *p*-value ≤ 0.001; # Nicotinate and nicotinamide metabolism (original KEGG pathway name); fMet—N-formylmethionine; ETC—electron transport chain; GSH—reduced glutathionine; log2 FC—fold-change on a log2 scale; NADH—reduced nicotinamide adenine dinucleotide; N-lac-AA—N-lactoylated-amino acids; TAG—triacylglycerol.

In the next approaches, samples were treated with either Rot, MPP or Ber for 24 h and compared for metabolome changes, based on an untargeted LC-MS-based method (Figure 1). In a PCA plot, all toxicant-exposed samples clearly separated away from the control, and also from one another, i.e., the replicates of a given toxicant clustered more closely together than the average values of the different mitotoxicants. However, Ber and MPP^+^ appeared closely related (Figure 7A). The differential metabolite abundance analysis confirmed the presence of multiple significant changes, with around 100 up- and 50–100 down-regulated metabolites per compound (Figure 7B). We directly compared MPP- and Ber-induced metabolic responses on a metabolite-by-metabolite level. A high correlation (R^2^ = 0.8) was observed (Figure 7C).

In a next step, we compared all toxicant-responses concerning the detailed metabolite changes taking place. The metabolites that were changed by at least two compounds were displayed in heat maps to visually check the similarity of metabolome responses (Figure 7D and Figure S10). A largely consistent pattern was observed. For instance, levels of lactate and its derivatives (N-lactoyl-AA [50] and indole-lactate) were greatly augmented by all three c-I inhibitors (Figure 7C,D). This confirms that the cells are highly glycolytic and that they used reducing equivalents (NADH) to convert the glycolysis end product pyruvate to lactate. This final reaction replenishes the NAD^+^ supplies, which are required to keep glycolysis running. This agrees with our data that NAD^+^ concentrations remained at control level (Supplementary Table S1), while NADH accumulated more than 16-fold (Figure 7C,D). The ratio NADH/NAD^+^ was therefore severely compromised, indicative of reductive stress.

We find also noteworthy that the Lys degradation products saccharopine and 2-aminoadipate were strongly increased. This points to a broad set of metabolic reverberations due to disturbances of primary metabolism. The disturbance of AA degradation observed here may be due to a stop of utilization in mitochondria. Importantly, the above intermediates have been implicated in triggering neuronal disease (epilepsy) [51]. Concerning modified amino acids, the acetylated AA were generally up-regulated, while dipeptides were down-regulated (Supplementary Figure S10). This pattern differs clearly from that produced by proteasome inhibitors, which down-regulated both groups of metabolites [11]. Energy and amino acid metabolism also play a key role in purine/pyrimidine metabolism. When we examined the group of nucleotide-related compounds, the nucleosides and purines/pyrimidines (e.g., guanine, cytidine, uridine, hypoxanthine and thymidine) were jointly up-regulated by mito-inhibitors, while other metabolites (e.g., various UDP-sugars) were consistently down-regulated (Figure S10). We suggest here as explanation that there was a lack of energy to convert the bases/nucleosides into energy-rich metabolites (GTP; UDP-glucose, etc.) used in biochemical transfer reactions. However, more detailed studies are required for clarification.

The most strongly down-regulated metabolite was 7-dehydrocholesterol. It is the last intermediate of cholesterol synthesis in the Kandutsch–Russel pathway. Inhibitors of this pathway severely impair brain development [52]. Notably, 7-dehydrocholesterol is the sterol that is most easily lost during lipid peroxidation, and its oxidative degradation product is neurotoxic [53]. We also examined whether the loss of 7-dehydrocholesterol only parallels a general loss of lipids, but cholesterol was much less down-regulated (Figure 7D). A clear up-regulation was observed for some ceramides related to triggering cell death (e.g., ceramide with two hexanoic acids [54,55]) (Figure 7D). This change may contribute to cell pathology. The ceramide precursors (various sphingomyelins) showed a heterogeneous regulation pattern (some up, some down), and most classical phospholipids were rather depleted (Figure S10). A detailed interpretation of the lipidomic pattern is not possible on the basis of the available data, but the observed regulations to both sides indicate that our sampling method did not create a technical artefact by generally enriching or depleting lipids.

Apart from this description of some conspicuous examples, we also applied unbiased characterization approaches. With an overrepresentation analysis (ORA) on the consensus metabolic signature, we checked for common pathway-level perturbations among the c-I inhibitors (Figure 7E). The top hits indicate alterations in the Warburg effect (glycolysis), the citric acid cycle (mitochondrial function) and AA metabolism (Arg and Pro, Gly and Ser, Asp, Glu). This is consistent with the initial conclusions from our biochemical analysis of amino acid levels and energy metabolism.

Even though a significance level of 5% was not reached (after correction for multiple testing), the degradation pathways for Lys and for branched chain amino acids (BCAA: Val, Leu and Ile) were enriched > 200% (Figure 7E). Some of the respective metabolites were individually up-regulated to a highly significant extent (Figure 7D). In all cases Ber followed the general trend of Rot and MPP. This was further followed-up below.

### 3.8. Global Transcriptomic Changes Induced by Mitochondrial Toxicants

**Figure 8:**
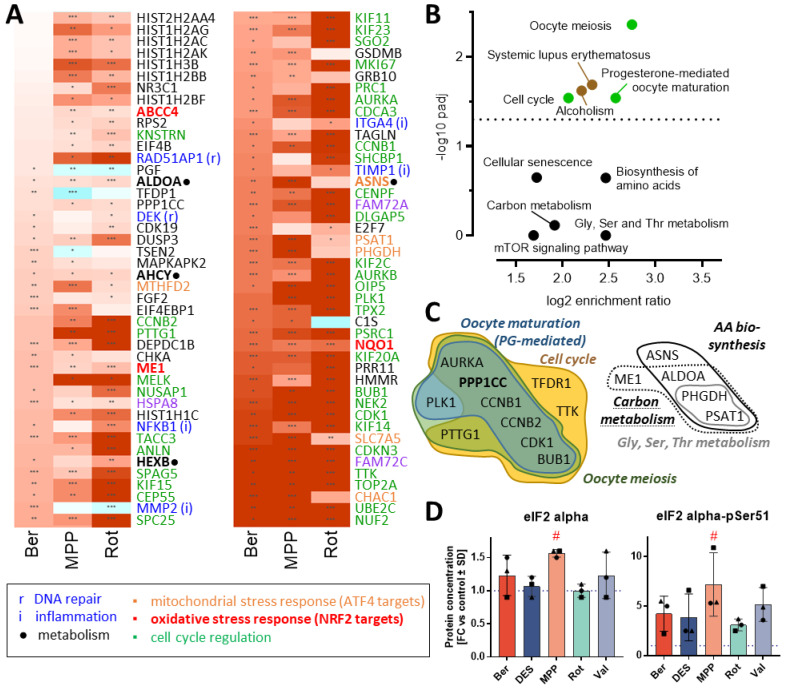
Consensus transcriptome changes induced by c-I inhibitors in neurons. Transcriptome data were obtained from LUHMES cells treated with berberine (Ber), rotenone (Rot) and MPP (MPP^+^) for 4 h, 16 h and 24 h, as outlined in Figure 1A. Differentially expressed genes were identified (see full details in Supplementary Table S1). For each compound and gene, the expression change was followed over time, and only the peak data (defined by significance) were kept as a dimensionality-reduced data set. Next, the genes with an adjusted *p*-value ≤ 0.05 and an absolute fold change (FC) > 1.5 (differentially expressed in ≥ two out of the three toxicant treatments) were selected for further analysis. (**A**) The up-regulated “consensus genes” (sorted in ascending order of the Ber FC values) are visualized in a heatmap. The color scale spans from blue (four-fold down) over white (no regulation) to red (four-fold up). Genes were mapped to functional categories, marked by different colors: cell cycle regulation and mitosis (green), mitochondrial stress response orchestrated by ATF4 (orange), oxidative stress response orchestrated by NRF2 (red-bold), inflammation (blue, i), DNA repair (blue, r), heat shock/toxicity response and disease/death pathways (purple) and carbon metabolism (black dot). The down-regulated “consensus genes” derived from the same analysis are separately displayed in Figure S11. (**B**) The biological pathways from the KEGG databank were analyzed for overrepresentation in the gene set shown in A. For the top 10 over-represented pathways, the significance (*y*-axis: -log2 adjusted *p*-value) was plotted against the enrichment ratio (*x*-axis). The dotted horizontal line marks the adjusted *p*-value of 0.05. (**C**) Venn diagrams of pathways from B) were used to display the relationships between the overrepresented gene sets. Not shown are the genes assigned to alcoholism (PPP1CC and histones) and systemic lupus (histones and C1S). (**D**) The levels of total eIF2 alpha protein and of phosphorylated eIF2 alpha-pSer51 were measured by a modified immunoblot (DigiWest). Individual samples are represented as dots; dot symbols indicate paired biological replicates. Data are means ± SD. Statistical analysis was performed vs. untreated controls (ANOVA followed by Dunnett’s post-hoc test); #: *p* < 0.05.

To obtain an additional layer of information, we re-visited the transcriptome data with a specific focus on the c-I inhibitors (Rot, MPP and Ber). We selected all regulated genes affected by at least two out of three compounds (Figure 8A and Figure S11). When the respective regulation peaks were aligned, we obtained a very consistent pattern of 88 genes (Figure 8A). The significantly enriched pathways were predominantly cell-cycle related (Figure 8B). A more detailed analysis showed that genes coding for cyclins (*CCNB2* and *CCNB1*), kinesins (*KIF15*, *KIF11*, *KIF23*, *KIF2C*, *KIF14* and *KIF20A*), spindle-assembly checkpoint proteins (*BUB1*, *CENPF* and *TPX2*) and cell cycle kinases (*CDK1*, *PLK1*, *AURKA* and *AURKB*) failed to be down-regulated (Figure 8C). A shift from cell differentiation towards continued proliferation (or de-differentiation) is a frequently observed, but unspecific response to toxicant stress. Also, the observed changes in histone expression are typical for cell stress during neurodegeneration [56]. Altogether, such relatively unspecific transcriptome regulations resulted here in a statistical over-representation of genes associated with systemic lupus erythematosus and alcoholism. We do not believe that this finding has a specific biological significance; it may rather indicate some general epigenetic re-arrangements triggered by many stressors. In this context, it is noteworthy that oxidative- and mitochondrial stress-related responses (expected for the reaction to mitotoxicants) did not feature among the pathway hits. This is a well-known issue of over-representation analysis in several cell types [12,57,58,59,60,61,62]. Mitotoxicants have a strong propensity to trigger cellular stress related to the transcription factors ATF4 and NRF2. However, the downstream response of the cells can be very complex, in that only subsets of the target genes are affected. A potential explanation is that a broad pattern of transcription factors may be activated, in parallel to multiple fast biochemical changes that affect the transcription machinery and that modify several regulatory feedback loops. Accordingly, our analysis clearly identified several important up-regulated ATF4 target genes (*MTHFD2*, *PSAT1* and *SLC7AC*) and also transcripts known to be under a strong control by NRF2 (*ABCC4*, *ME1* and *NQO1*). In previous work, we found the translation controller eIF2 alpha to be phosphorylated in MPP-exposed LUHMES neurons [10]. Phospho-eIF2 alpha is a major activator of ATF4, and we confirmed here our previous observation. Most other toxicants led to average increases of about 400% (Figure 8D). This trend (not statistically significant) suggests that the ATF4 pathway is indeed activated, yet several other analysis methods and a better time resolution would be required to study the signal pathway in detail.

For discussion of the above observations, it is interesting that our over-representation analyses did not indicate pathways/ontologies involved in DNA repair and inflammation (which would also be expected). One potential explanation is the overwhelming contribution of the cell cycle genes to the consensus signature, and thus a reduction in the over-representation significance for other gene groups. This is an inherent weakness of over-representation analysis. Its detailed clarification is beyond the scope of our work, but our findings provide an example for the need to consider new forms of analysis, e.g., excluding less specific regulations to unmask hidden specific changes. Indeed, a clear signal for changes in AA metabolism was derived from the transcriptome data (pathway enrichment factors of up to 500%), but the adjusted significances remained low (Figure 8B). This may be different, if a filtered subset of metabolism-related genes was analyzed.

Our data also suggest that a combination of metabolomics and transcriptomics can increase the specificity of toxicological information, compared to conventional transcriptome studies. Indeed, many toxicants can lead to pronounced, rapid and specific metabolome changes that are not necessarily followed by corresponding transcriptome regulations (e.g., when the cell dies or when the energy status is too low, or when oxidative stress destroys key enzymes required). In such cases, transcriptome analysis is less sensitive and possibly less specific (indicating general stress, but not a mode of action of toxicants). Vice versa, other toxicants (e.g., epigenetic modifiers) may change the transcriptome with little biochemical change [63].

### 3.9. Far-Reaching Changes in AA Metabolism, Exemplified by Lys Degradation

The transcriptome analysis and metabolome analysis indicated changes in amino acid metabolism (Figure 7E), and this agreed well with our pilot measurements, which detected changes in the levels of amino acids themselves. To explore whether there is a more generalized pattern behind the list of altered metabolites, we used a “biased” approach (different from the statistics-based over-representation analyses): we compiled all changes that could be related to amino acid metabolism according to text book knowledge.

**Figure 9:**
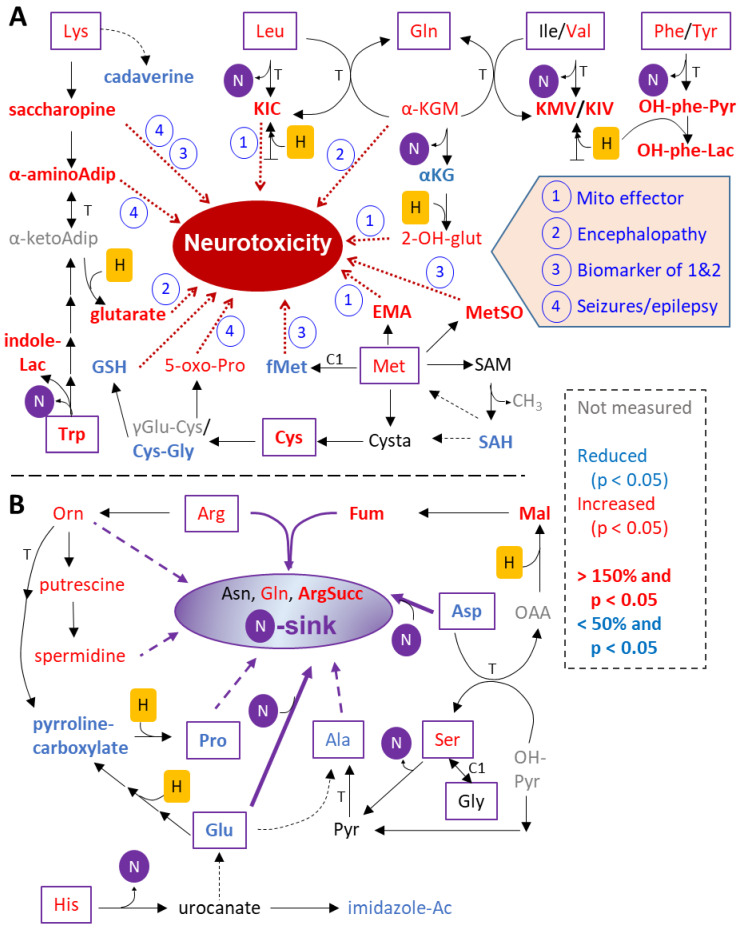
Overview of the changes related to the neuronal amino acid metabolism after exposure to c-I inhibitors. The scheme was constructed to visualize potential connections of the altered amino acid metabolism with neurotoxicity (dotted red arrows), with nitrogen elimination (purple, “N”) and with disposal of excess reducing equivalents (yellow, “H”). The metabolites that are indicated were measured after 24 h incubation of LUHMES cells with berberine chloride (12.5 µM, 4 independent experiments). Boxed compounds are proteinogenic amino acids. Red labelling indicates a significant cellular accumulation, blue labelling a depletion (adjusted *p* < 0.05); black indicates metabolites that were not altered significantly. Metabolites which were not quantified (but help to understand the metabolic map), are depicted in gray. All metabolites that changed > 1.5 fold are marked by “bold” formatting. Essentially similar data were obtained for Rot (0.5 µM) and MPP (10 µM). Details are found in Supplementary Table S1. (**A**) Contextualization of reactions and metabolites directly related to neurotoxicity. (**B**) Focus on the cellular need to dispose of nitrogen. Exemplary compounds that incorporate nitrogen from other amino acids, and that therefore can act as intermediate “nitrogen sink” (or as export vehicles) are shown. Reactions in (**A**) labelled with “N” would end up in the same nitrogen sink. α-aminoAdip—2-aminoadipate; α-kB—α-ketobutyrate; α-ketoAdip—α-ketoadipate; α-KG—α-ketoglutarate; α-KGM—α-ketoglutaramate; ArgSucc—argininosuccinate; C1—1-carbon metabolism; Cysta—cystathionine; EMA—ethylmalonate; fMet—N-formylmethionine; Fum—fumarate; “H”—NAD(P)H; imidazole-Ac—4-imidazoleacetate; indoleLac—indolelactate; KIC—α-keto-Leu (keto-isocaproate); KIV—α-keto-Val (keto-isovalerate); KMV—α-keto-Ile (keto-methylvalerate); OH-phe-Lac—3-(4-hydroxyphenyl)lactate; OH-phe-Pyr—4-hydroxyphenylpyruvate; 2-OH-glut—2-hydroxyglutarate; 5-oxo-Pro—5-oxoproline; Mal—malate; MetSO—methionine sulfoxide; “N”—-NH3; Orn—ornithine; SAH—S-adenosylhomocysteine; SAM—S-adenosylmethionine; Succ-CoA—succinyl-coenzyme A; T—transaminase.

Twelve of the twenty proteinogenic amino acids were up-regulated by Ber, five were down-regulated and only Gly, Ile and Thr were not altered (Figure 9A,B). Similar data were obtained for MPP and Rot (Supplementary Table S1, Figure 7D). As the patterns of MPP, Rot and Ber resembled one another, and as Ber is the compound least characterized yet, all following data refer to this toxicant.

On first sight, a consistent metabolite up-regulation in a group of related compounds is surprising in cells exposed to metabolic inhibitors. However, metabolism is a network with some major hubs (e.g., the tricarboxylic acid (TCA) cycle). Block in a hub may lead to (i) backwater in metabolic pathways, and (ii) to re-routing into pathways normally used less. Both effects can lead to metabolite accumulations. Indeed, the degradation of AA ends in many cases in metabolites that are transferred into the TCA. As mitotoxicants lead to a disturbance of the TCA, a backwater in AA metabolism may be expected [64]. To check this, we examined changes in AA degradation products. Some exemplary findings are discussed below.

Lys degradation produces several breakdown intermediates, many of which significantly accumulated in the c-I-inhibited neurons: saccharopine, α-aminoadipate and glutarate (Figure 9A). In LUHMES cells with impaired c-I function, we observed a > 40-fold increase in saccharopine, which was by far the most up-regulated metabolite measured here. Degradation of Lys via the saccharopine pathway predominantly takes place in the mitochondria of cultured human brain cells [65]. The increased saccharopine levels following c-I inhibition is not a unique observation to neurons; HeLa cells treated with piericidin are also enriched in saccharopine and localized predominantly in the mitochondria [66]. The accumulation of saccharopine in urine is a biomarker of an aminoacidopathy (saccharopinuria) (Figure S12), which—if left untreated—can lead to brain dysfunction [67]. Furthermore, saccharopine excess was recently shown to impair mitochondrial dynamics and function [68].

Further downstream in the Lys breakdown reaction chain, glutarate was also ≥ six-fold up-regulated. Glutarate is a biomarker of another inborn error of metabolism called glutaric aciduria type I (Figure S12), linked to neurological disabilities [69]. The pathway towards glutarate is favored by an abundance of NADH, as in the presence of c-I inhibitors (need for reduction in alpha-keto-adipate). The other major pathway of lysine catabolism, the “pipecolic acid pathway” was affected in a different way: its first intermediate, cadaverine, was significantly decreased by all three c-I inhibitors. The Lys degradation connects to Trp degradation via α-aminoadipate and its metabolite α-aminoadipic semialdehyde. Metabolic effects relating to the latter have been linked to human epilepsies [65].

The example of Lys-related pathways indicates already two possibly general principles: (i) some of the up-regulated metabolites are related to human neurological disease or to neurodegeneration. The alterations in amino acid metabolism may not only serve a biomarker function, but they may be directly involved in the cell pathology (in addition to direct effects of c-I inhibitors on mitochondria); (ii) the abundance of NADH might drive some reactions, i.e., shift reaction balances in a way not found in healthy cells.

### 3.10. Altered Branched Chain AA Metabolism as Secondary Consequence of Mitochondrial Inhibition

The first reaction in the catabolism of branched chain amino acids (BCAA: Leu, Ile, Va) is their irreversible transamination, yielding the corresponding branched chain ketoacids (BCKA: KIC, KIM and KIV) (Figure 9A). These intermediates are strongly up-regulated by all three c-I inhibitors (Figure 7D). The accumulation of BCKA serves as a biomarker for maple syrup urine disease (MSUD) [70]. Such an accumulation happens when the next reaction cannot occur, due to mutations in the enzyme catalyzing it (BCKDH). Left untreated, MSUD can lead to encephalopathy, including epileptic seizures. The metabolites themselves (e.g., alpha-ketoisocaproic acid (KIC)) are neurotoxic and inhibit mitochondrial respiration [71] (Figure S12).

A general theme of AA catabolism is that the amino-nitrogen needs to be transferred to another compound for further disposal. A known amino-acceptor for BCAA is α-ketoglutarate (α-KG); an alternative acceptor (in a reaction catalyzed by a glutamine transaminase) is Gln, which forms α-ketoglutaramate (α-KGM) [72] (Figure 9A). The levels of α-KGM were increased (Figure 7D). In a second reaction step (belonging to the cytosolic glutaminase II pathway), α-KGM is hydrolized by ω-amidase (Nit2) to form ammonia and α-ketoglutarate (α-KG). Notably, under conditions of NADH excess (c-I inhibition), α-KG is metabolized to 2-hydroxyglutarate (2-OH-glut) by several dehydrogenases (LDH, MDH and PHGDH) [73]. There is evidence that this pathway was indeed activated by Ber, as α-KG was decreased and 2-OH-glut was augmented. This is significant for the mechanism of neurotoxicity, as 2-OH-glut accumulation was recently discovered to be at the core of 2-hydroxyglutaric aciduria, a disease with several neurological symptoms [73]. Likewise, α-KGM is increased in the cerebrospinal fluid of patients with neurological symptoms, such as (hepatic) encephalopathy [72,74] (Figure S12). The above suggested general learnings seem to be confirmed by the example of BCAA.

### 3.11. Novel Stress Markers Due to Altered AA Metabolism

One of our most intriguing findings was the severe reduction (down to less than 10%) in N-formyl-Met (fMet) (Figure 7D). This modified amino acid plays a unique role in translation initiation of proteins encoded by the mitochondrial DNA. The reduced fMet levels may make mitochondrial protein synthesis impossible, and this would contribute to a perturbed homeostasis of mitochondrial proteins upon prolonged toxicant exposure. The finding is particularly significant in the light of our data that non-modified Met as such was not reduced, and thus available within the cells.

We also found the oxidative stress marker, 5-oxo-Pro to be augmented (Figure 7D and Figure 9A). This was consistent with a depletion of GSH (down to less than 25%) and of its precursors γ-Glu-Gly (down to ~ 15%) and Cys-Gly (Figure 9A). High levels of 5-oxo-Pro in urine are biomarkers for damage to the central nervous system [75].

### 3.12. Need for a Nitrogen Sink to Allow Altered Metabolism of AA

While AA contain 1-2 nitrogen atoms, many of their metabolites do not. Nitrogen must thus be bound organically somewhere to prevent the release of ammonia (NH_3_). NH_3_ is a well-studied neurotoxicant whose accumulation can lead to brain edema [76]. Nitrogen sinks, molecules that act as nitrogen acceptors, are therefore important for toxicant-altered metabolic states of neurons (Figure 9B). In some cell types (hepatocytes and astrocytes) a complete urea cycle takes an important role in this. In neurons, urea synthesis is limited, but some of the reactions are possible. For instance, argininosuccinate production (a key step of the urea cycle) took place and was increased (200%). Also, the levels of the N-rich AA Arg and Orn were increased. Moreover, the levels of the polyamines putrescine and spermidine, formed by Orn decarboxylation were augmented. Since polyamine metabolism regulates cellular homeostasis on many levels, any perturbation may be of concern.

Amongst the AA, Asn and Gln can act as nitrogen sinks. They can be synthesized from Asp and Glu, and indeed, Glu and Asp were highly depleted. Glu is metabolically related to His, as His can be metabolized to Glu via the intermediate urocanate. In Ber-treated neurons His and urocanate (n.s.) levels were at >200% of normal. Whether this is due to an altered level of one of the other AA is at the moment speculative (flux data would be required). However, the up-regulation of yet another metabolite linked to intellectual disability (urocanic aciduria) [77] fits the general picture of a pathologically altered metabolic pattern far beyond what would be considered mitochondrial pathways.

### 3.13. Krebs Cycle (TCA) Perturbation

**Figure 10:**
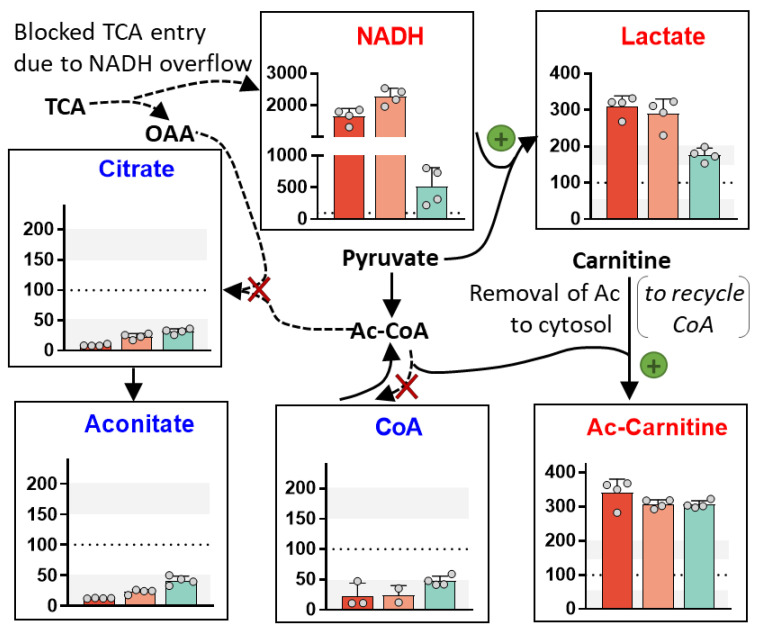
Altered TCA entry scenario upon c-I inhibition. LUHMES cells were exposed to three c-I inhibitors at equipotent concentration for 24 h: 12.5 µM berberine (red), 10 µM MPP^+^ (light red) and 0.5 µM rotenone (blue). The bar graphs show the percent changes of the respective metabolites (compared to solvent controls). Data are means and individual data from four independent experiments. The arrows indicate the underlying metabolic pathways. The red crosses indicate reactions that seem to be blocked in the presence of c-I inhibitors. The green pluses indicate reactions that seem to be enhanced in the presence of c-I inhibitors. The tree inhibitors trigger “similar” changes, but the typical glycolytic enhancements (NADH and lactate up) are quantitatively different. CoA—coenzyme A; Ac—acetyl; OAA—oxaloacetic acid; TCA—tricarboxylic acid cycle.

We examined here the size of several metabolite pools clearly linked to TCA turnover (Figure 10). Citrate and aconitate were depleted by >50% by the three c-I inhibitors. The amount of citric acid in the TCA correlates with the overall flux through the canonical TCA (speed of the oxidative/catabolic version of the TCA). The aconitate levels were closely correlated and confirmed the citrate data. In parallel, we observed a depletion of free CoA to <50% of control values, which indicates also a block of the TCA. We also found that all c-I inhibitors caused a massive accumulation of Ac-carnitine (300%), a typical side pathway activated upon TCA inhibition.

The c-I inhibition also lead to a massive accumulation of NADH in the cell and an increased formation of lactate. These changes are canonical signs of a reduced TCA flux.

### 3.14. Evidence for Metabolic Re-Routing as Consequence of c-I Inhibition

**Figure 11:**
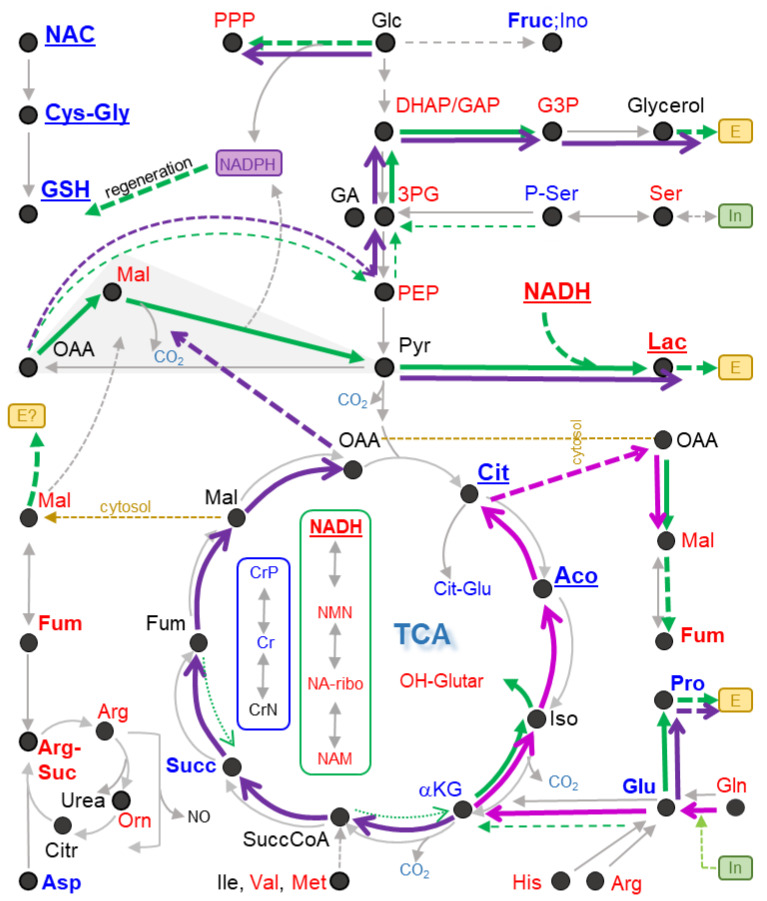
Shift of the primary metabolism of neurons by mitochondrial inhibitors. LUHMES cells (d2) were exposed to 12.5 µM berberine (Ber), 10 µM MPP^+^ (MPP) or 0.5 µM rotenone (Rot) for 24 h before intracellular metabolites were extracted, analyzed and quantified. The data displayed refer to the treatment with Ber. Data on MPP and Rot are found in Supplementary Table S1, and their pattern was largely similar to that shown for Ber. The grey arrows depict standard/canonical cellular metabolism, such as the oxidative tricarboxylic acid (TCA) cycle (bottom), and glycolysis feeding it (top). Strict compartmental separations of pathways are not indicated, as the analysis approach used did not separate compartments. However, the upper part of the diagram represents mainly cytosolic processes, the TCA reactions are in mitochondria, and some of its metabolites are indicated twice in order to make clear that they are also found in the cytosol (transitions indicated in yellow), and may have different concentrations in each compartment. Color codes were used for compounds that were significantly (adjusted *p*-value ≤ 0.05) up-regulated (red) or down-regulated (blue). Compounds that were not quantified, or for which the concentration was unclear (e.g., mitochondrial malate) are shown in black (the total cellular concentration of malate is indicated elsewhere). Strong and significant regulations (fold change ≥ 2) are marked in bold and very strong changes (fold change ≥ 3) are underlined. Green arrows indicate pathways that get rid of excess reducing equivalents (NADH). Dashed versions indicate reactions leading to these reactions or following from such reactions. Dark pink arrows indicate the pathway of “reductive carboxylation”, which allows conversion of glutamine to citrate and malate/fumarate by using NADH (instead of generating NADH). The purple arrows indicate hypothetical pathways that we suggest to be activated under conditions of electron transfer chain inhibition. The grey triangle indicates reactions performed by a “cytosolic hydride transfer complex” that regenerates NAD+ from NADH. “E/In” indicates a potential import or export across the cell membrane. Three isolated groups of compounds (not linked here to the carbon metabolism network) shown refer to: GSH and its precursors, NADH and its metabolites and the mitochondrial energy buffer creatine phosphate and its metabolites. Dotted green arrows indicate reactions using up reducing equivalents, but that were considered unlikely to take place. 3PG—3-phosphoglycerate; Aco—aconitate; Arg-Suc—argininosuccinate; Cit—citrate; Cit-Glu—citrylglutamate; Citr—citrulline; Cr—creatine; CrN—creatinine; CrP—creatine phosphate; Cys-Gly—cysteinyl-glycine; DHAP: dihydroxyacetonephosphate; Fruc—fructose; Fum—fumarate; G3P—glycerol-3-phosphate; GA: glyceraldehyde; GAP—glyceraldehyde phosphate; GSH—glutathione reduced; Glc—glucose; Ino—myo-inositol; Iso—isocitrate; Lac—lactate; Mal—malate; NA-riboR—nicotinamide-riboside; NAC—N-acetylcysteine; NAD(P)H—reduced nicotinamide adenine dinucleotide (phosphate); NAM—nicotinamide; NMN—nicotinamide ribonucleotide; NO—nitric oxide; OAA—oxaloacetate; OH-Glutar—2-hydroxyglutarate; Orn—ornithine; P-Ser—phosphorylserine; PEP—phosphoenolpyruvate; PPP—pentose phosphate pathway—represented by erythronate; Pyr—pyruvate; Succ—succinate; SuccCoA—succinyl-CoA; αKG—α-ketoglutarate.

The metabolic consequences of c-I inhibition are extremely well-studied in cancer cells. Less information is available for neurons, but there is evidence that some of the effects would be conserved [10,78,79,80]. For instance, it has been suggested that enzymatic reactions known for the TCA may still occur, but they may be reversed or may take a role in feeding alternative pathways [81,82,83,84,85]. The immediate effect of c-I inhibition is that NADH is less consumed in the ETC. In parallel, less NAD+ is produced; it lacks therefore as a substrate of dehydrogenase reactions. This “reductive stress” (= increased NADH/NAD^+^ ratio) leads to a reversal of several metabolite balances. Moreover, the metabolites directly related to NADH are likely to be increased. Indeed, we observed the up-regulation of three NADH metabolites, and we observed strong (indirect) evidence for directions in the TCA (Figure 11).

We suggest that neurons treated with Ber switched to reductive carboxylation. In this reaction, cells can utilize Gln in the absence of an ETC and under conditions of NADH overload. They use the “reversed right side of the TCA” to introduce CO_2_ to the C5-carbon chain of Gln in order to generate citrate (C6 chain). Citrate, exported to the cytosol, can be used to generate Ac-CoA for lipid synthesis and OAA plus NADH to generate malate. Malate is part of the recently discovered “hydride transfer complex” reaction [86]. The latter dissipates excessive NADH, supplies missing NAD^+^ and generates NADPH, an essential electron donor to counteract oxidative stress (regeneration of GSH) and to support lipid synthesis. In support of this hypothesis, we observed an accumulation of malate (and of the related metabolite fumarate), as well as OH-glutarate, a normally rare mitochondrial metabolite generated during reductive stress and as by-product of reductive carboxylation.

Glutamine may also be converted towards malate by glutaminolysis, i.e., by a sequence of reactions using only “the left part of the TCA”, not involving citrate synthesis. This is a well-known pathway in cancer cells. Whether it occurs in c-I-inhibited neurons depends on the extent that the succinate dehydrogenase reaction and the αKG-dehydrogenase reaction would still be working.

In the absence of a detailed isotope flux analysis, it is not possible to confirm that glycolysis is indeed running at an increased speed. The cellular need for ATP argues for a high glycolytic rate, while the overload with NADH rather suggests that glycolysis should be reduced. At present, several hypotheses need to be considered to explain the observed metabolite changes; while the high levels of NADH and lactate suggest that the last reaction of anaerobic glycolysis runs at a high level, the pyruvate needed for lactate formation may be derived from glycolysis or from other sources (e.g., alanine or OAA). Similarly, our data on glucose consumption (Figure 4) do not prove that canonical glycolysis is running. Glucose may also be utilized to a large extent to support the pentose phosphate pathway (PPP) for generation of NADPH. Moreover, glucose may be converted to fructose/sorbitol or myo-inositol (Figure 11). The latter metabolites were found to be down-regulated here. But this may be due to an increased export from cells. Indeed, several metabolites found here to be down-regulated within cells were found to be increased in the plasma of patients with a mitochondrial disease (sorbitol, myo-inositol, aspartate, alanine, creatine or cystationine (S-adenosylhomocysteine as proxy)) [87]. The relationship between intracellular levels and what is found in plasma will need further investigation.

The up-regulation of several intermediates (DHAP, PEP and GAP, see Figure 11) may indicate that glycolysis is up-regulated. An alternative explanation would be that C4 bodies (e.g., malate/OAA) generated from AA may be converted to C3 bodies (glycerol) to be excreted. Such a reaction sequence would dissipate NADH, which is present in excessive amounts in c-I inhibited neurons.

Altogether, this last part of our study clearly shows that massive metabolic changes occur, even while cells maintain their ATP levels. An energetic stress may only be indirectly deduced, by the depletion of phospho-creatine (Creatine-P) (Figure 11). Creatine-P is the most important energy buffer of mitochondria. Its loss may make them more sensitive, but the metabolic changes we analyzed all occur in cells still viable and still able to retain high ATP levels. From this, we conclude that the altered behavior of the TCA and the reductive state of neurons (high NADH) may be major drivers of the metabolic derangement. We showed severe consequences on AA and their metabolites (Asp most down-regulated; Lys metabolites most up-regulated). Notably, we found many lactoyl-AA up-regulated, which agrees well with findings from patients with a mitochondrial disease [88]. Thus, our study definitely shows that c-I inhibition triggers a wide-spread metabolic re-programming. Moreover, many of the metabolite changes may contribute to neuronal dysfunction and eventually to neurodegeneration, even if cells are not starved of ATP. Thus, the metabolic shifts observed here may contribute to, or even drive, the long-term adverse effects occurring after mild c-I inhibition over a long time.

## 4. Conclusions

Our study followed up on seven hits from a (developmental) neurotoxicity screen [4] and particularly focused on an in depth description of metabolic disturbances triggered by Ber, one of the three c-I inhibitors originally identified as hits. Three major objectives were followed in this study: (i) From a toxicological point of view, we wanted to understand better the neuronal consequences of c-I inhibition. We provided here novel data on a plethora of secondary metabolic changes. Many of them may contribute to pathology, in particular during chronic exposure, i.e., situations, where c-I inhibition is not killing cells simply by ATP-depletion. (ii) From a drug screening point of view, we explored a strategy to follow up on hits from phenotypic screens to better understand, on a mechanistic basis, why they emerged as hits, and whether the finding is of toxicological relevance. While transcriptomics alone may not yield sufficient specific information on some toxicants, a multi-omics approach was here more useful, at least for some compounds. (iii) Detailed information on Ber’s mode of action, in comparison to archetypical c-I inhibitors, was obtained on the transcriptome and metabolome level. The redox-active compound was considered of high interest, as it emerged recently as a nutraceutical ingredient of food supplements meant to counteract aging and several diseases [89,90,91,92,93,94].

Most of the novel data were provided by the metabolomics approach. We observed not only the “trivial” endpoints, such as a dramatic increase in NADH and a decrease in citrate/aconitate. Rather, we found evidence for complex metabolic re-routing (altered AA metabolism; generation of Ac-carnitine, etc.). Given this rich set of data, we consider it possible that Ber, even if fully unknown at the onset of the study, may have been identified as c-I inhibitor by this approach.

Another conclusion from this study is that the interpretation of a mode-of-action is clearly strengthened by the comparison to known targets. We conclude that the most robust approach to the elucidation of the mechanism of an unknown toxicant would be to identify data-rich compounds with a supposedly similar mode-of-action and then to compare the compounds within the given experimental model face-to-face. Such approaches, called read-across strategies [95,96,97], may not always be feasible, but this situation is going to change as more data become available on a broad range of compounds (e.g., by large-scale screening programs). It will likely be possible in the near future to mechanistically anchor unknown chemicals to known toxicants [98,99].

The up-regulation of a whole panel of metabolites known to be associated with neurodegeneration and with mitochondrial defects (saccharopine, α-aminoadipate, glutarate, α-ketoisocaproate (KIC), α-ketoglutaramate (α-KGM), 2-hydroxy-glutarate (2-HG), ethylmalonate (EMA) and N-formyl-methionine (fMet)) was particularly evident in our study. This occurred at toxicant concentrations that did not deplete ATP. Thus, mitochondrial inhibitors may act through mitotoxic endogenous metabolites that accumulate in neurons under such conditions. To our knowledge, this hypothesis is novel and deserves further investigation. An interesting starting point for this may be further studies on the role of KIC (and other branched chain AA metabolites), as KIC is found in neurodegenerative disorders, and it has been suggested to impair mitochondrial functions [71]. Other candidates, little noticed and explored yet, are metabolites of the aromatic AA Tyr and Phe. OH-phenyl-pyruvate was increased by >60% in Ber and MPP treatments; OH-phenyl-lactate by more than two-fold by all three c-I inhibitors. High levels of phenolic metabolites in urine are a hallmark of tyrosinemia type III, a rare inherited disorder associated with neurological symptoms [100].

One overarching feature of metabolic changes observed was reductive stress. In particular hydroxy-glutarate (OH-glutarate; found here up-regulated) is considered a specific biomarker for reductive stress within mitochondria [101]. This is more specific than NADH (whose origin and cellular localization are hard to determine from standard metabolomics data). The up-regulation of OH-glutarate also suggests that reduced levels of, e.g., citrate, are unlikely due to an overall loss of mitochondrial mass. In the future, it may become possible to analyze the metabolome of cellular sub-compartments, such as the mitochondrial matrix [66]. This may lead to an increased sensitivity, as, e.g., the rise of saccharopine levels or the depletion of aspartate upon c-I inhibition is stronger in the mitochondrial matrix than in whole cells [66]. However, in our cell model, the extent of metabolite change measured was very high, compared to many metabolome studies, and many large and significant regulations were observed without the need for subcellular sampling.

While reducing equivalents (NADH) were strongly up-regulated and drove some of the overall metabolic shifts (a situation termed reductive stress), we also found evidence of oxidative stress (e.g., loss of glutathione or up-regulation of the transcript for NQO1). This is only apparently a contradiction. Indeed, reductive stress contributes to an increased production of reactive oxygen species in various direct and indirect ways. Best known is the reduction of oxygen to superoxide and hydrogen peroxide. Also well-known is the alteration of cellular iron pools and redox-states, which makes more ferrous iron available to participate in the Fenton reaction [102]. Alternatively, the labile iron pool may be increased. This again may contribute to cell death processes such as ferroptosis [103,104]. This iron release has been found to be important for dopaminergic neuron death and differentially affected by various toxicants [105].

One important future question, not addressed here in detail, are differences in the effects of c-I inhibitors. Such differences have been observed repeatedly on a cellular level [12]. One explanation may be differences in affinity and cellular distribution. Thereby, the ratio of ROS formation and ETC inhibition may be slightly altered, and our study shows that this may be reflected in a large panel of secondary metabolic consequences, that themselves may again affect mitochondrial function or other cellular reactions. An alternative explanation may be the existence of additional cellular targets, as exemplified here by the microtubule polymerization data. There is a large need for further studies on the metabolic changes beyond the evident primary target reactions (citric acid cycle and its entry pathways). Notably, also a highly sophisticated study on mitochondrial matrix metabolomics ended with such a conclusion, and explanations on some puzzling findings were not evident. For instance, various ETC inhibitors triggered quite diverse metabolite responses [66].

On the level of the whole organism (rats or humans), some differences may be easier to explain. While MPP and Ber were found to have very similar effects on neurons here, MPP is a parkinsonian neurotoxin in vivo [106], while Ber has been used to treat, e.g., type II diabetes [107]. The likely explanation at present is a very low bioavailability of Ber, and a strong hepatic metabolism that eliminates the toxicant [108,109]. In view of Ber’s use as nutraceutical, and of the findings that it may act as potent cytotoxicant, some more detailed toxicological studies seem advisory. Physicochemical data suggest that Ber would cross many barriers (intestinal, blood-brain) at high efficiency (Supplementary Figure S13). In this context, an important future study should evaluate whether the metabolic changes triggered by Ber are specific to neurons. After oral uptake, the first type of cells in contact with Ber are the lining cells of the gastro-intestinal tract. Even though Ber might have a high first pass, it has to be assumed that, e.g., the compound would penetrate the membrane of enterocytes. Moreover, it is likely that the compound would also pass the intestinal lining to reach the portal blood. Even if it was all eliminated in the liver, one would expect that cells lining the hepatic sinusoid would be exposed to Ber. A comparison of secondary metabolic re-programming by Ber and other c-I inhibitors between neurons and other cell types (e.g., hepatocytes or enterocytes) would produce useful data sets. These would help to answer the question about how far some of the amino acid metabolites described here are specifically associated with neurotoxicity, or whether they are also observed in cells that are not affected in their viability by Ber.

## Data Availability

The omics data presented in this study are available in Supplementary Table S1.

## Supplementary Materials

The following supporting information can be downloaded at: https://www.mdpi.com/article/10.3390/antiox13010049/s1, Figure S1: Mechanistic description of the 7 DNT assay hits; Figure S2: Overview of de-regulated genes over time; Figure S3: Display of transcription factors with the highest predicted activity scores at 16 h; Figure S4: Activity predictions for transcription factor ATF4; Figure S5: Expression of consensus deregulated genes at 4, 16 and 24 h; Figure S6: Display of genes with highest down-regulation at 16 h; Figure S7: Effect of toxicants on neuronal ATP production; Figure S8: Effect of toxicants on microtubule polymerization; Figure S9: Intracellular amino acid changes in immature neurons exposed to neurodevelopmental toxicants; Figure S10: Other metabolic changes induced by c-I inhibitors in neurons; Figure S11: Consensus transcriptome changes induced by c-I inhibitors in neurons; Figure S12: Metabolites linked to aminoacidopathies; Figure S13: Pharmacokinetic parameters of berberine; Suppl. Results: Detailed results for sections 3.2, 3.6, 3.11 and 3.13; Table S1: Statistical results of omics experiments. References [110,111,112,113,114,115,116,117,118,119,120,121,122,123,124,125,126,127,128,129,130,131,132,133,134,135,136,137,138,139,140,141,142,143] are cited in the supplementary materials.
