# Supplementary material for: Definition of the Neurotoxicity-Associated Metabolic Signature Triggered by Berberine and Other Respiratory Chain Inhibitors
Source: Antioxidants (Basel). 2023 Dec 28;13(1):49. doi: 10.3390/antiox13010049 (PMC10812665; doi:10.3390/antiox13010049)
Supplement: Supplementary file 1 [file antioxidants-13-00049-s001.zip › Berberine Suppl Fig and Results.pdf]

Supplementary Material

Definition of the Neurotoxicity-Associated Metabolic Signature Triggered by Berberine and Other Respiratory Chain Inhibitors

Ilinca Suciu[1,2], Johannes Delp[1], Simon Gutbier[1], Julian Suess[1], Lars Henschke[2,3], Ivana Celardo[1], Thomas U. Mayer[3], Ivano Amelio[4], Marcel Leist[1,*]

1 In Vitro Toxicology and Biomedicine, Department Inaugurated by the Doerenkamp-Zbinden Foundation, University of Konstanz, 78464 Konstanz, Germany
2 Graduate School of Chemical Biology, University of Konstanz, 78464 Konstanz, Germany
3 Department of Molecular Genetics, University of Konstanz, 78464 Konstanz, Germany
4 Division for Systems Toxicology, Department of Biology, University of Konstanz, 78464 Konstanz, Germany
* Correspondence: marcel.leist@uni-konstanz.de

Table of Contents

Element	Page	Title
Fig. S1	2 - 3	**Mechanistic description of the 7 DNT assay hits**
Fig. S2	4 - 5	**Overview of de-regulated genes over time**
Fig. S3	6 - 7	**Display of transcription factors with the highest predicted activity scores at 16 h**
Fig. S4	8 - 9	**Activity predictions for transcription factor ATF4**
Fig. S5	10 - 11	**Expression of consensus deregulated genes at 4, 16 and 24 h**
Fig. S6	12	**Display of genes with highest down-regulation at 16 h**
Fig. S7	13	**Effect of toxicants on neuronal ATP production**
Fig. S8	14	**Effect of toxicants on microtubule polymerization**
Fig. S9	15	**Intracellular amino acid changes in immature neurons exposed to neurodevelopmental toxicants**
Fig. S10	16	**Other metabolic changes induced by c-I inhibitors in neurons**
Fig. S11	17	**Consensus transcriptome changes induced by c-I inhibitors in neurons**
Fig. S12	18	**Metabolites linked to aminoacidopathies**
Fig. S13	19 - 20	**Pharmacokinetic parameters of berberine**
Suppl. Results	21 - 24	**Detailed results for sections 3.2, 3.6, 3.11 and 3.13**
Bibliography	24 - 27	
Table S1		**Statistical results of omics experiments** (separate file)

**Berberine** (Cas: 2086-83-1) is a plant alkaloid with several applications in alternative and traditional medicine. It is for example used as an anti-diabetic agent and considered to promote longevity. Berberine inhibits mitochondrial respiration and promotes glycolysis. An inhibition of mitochondrial complex I inhibition, has been reported in e.g (1) primary mouse hepatocytes (IC50: ca. 5 µM, Zhang et al. 2018), (2) in primary rat muscle cells (IC50: ca. 15 µM, Turner et al. 2008) and (3) in a human dopaminergic neurons (IC50: ca. 20 µM; Delp et al. 2019). In cell painting assays, berberine affects mitochondria-related parameters at 1-10 µM (Nyffeler et al. 2020). Berberine has also been suggested to inhibit acetylcholinesterase (IC50: 2 µM, Li et al. 2021). The compound has very low oral bioavailability in rats (<0.3%). It is rapidly and extensively metabolized in the liver (Turner et al. 2008).		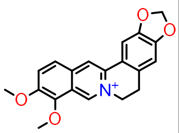
MW [g/mol]: 336.4	LogP: 0.82*	SMILES: COC1=C(C2=C[N+]3=C(C=C2C=C1)C4=CC5=C(C=C4CC3) OCO5)OC

**Carbaryl** (Cas: 63-25-2) is a broad-spectrum insecticide belonging to the class of carbamates. It inhibits human acetylcholinesterase with a reported IC50 of 2 µM (Li et al. 2021). Thus, carbaryl is neurotoxic. It is also known for developmental neurotoxicity, and it might be carcinogenic due to its effect on cell cycle and chromosome separation. In the Toxcast screening, it affected several assays, including neuronal network activity (2 µM) and inhibition of GLI family zinc finger 3 (GLI3 – IC50: 13 µM).		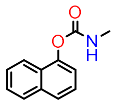
MW [g/mol]: 201.2	LogP: 2.36	SMILES: CNC(=O)OC1=C2C=CC=CC2=CC=C1

**Colchicine** (Cas: 64-86-8) is a plant alkaloid used for several medical applications. It inhibits tubulin polymerization and thereby impairs neurite transport and mitotic chromosome segregation. Tubulin polymerization inhibition data are available for pig brain tubulin (IC50: 7 nM, Thomopoulou et al. 2016) and bovine tubulin (IC50: 11 nM, El-Naggar et al. 2020). Furthermore, it induces neuronal apoptosis at 0.1 - 1 µM (Leist et al. 1997; Volbracht et al. 1999).		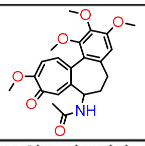
MW [g/mol]: 399.4	LogP: 1.30	SMILES:COC1=CC2=C(C(OC)=C1OC)C1=CC=C(OC)C(=O)C=C1[C@H](CC2)NC(C)=O

**Diethylstilbestrol** (Cas: 56-53-1) is a synthetic nonsteroidal estrogen receptor agonist banned because of its teratogenic effects. Reported potencies for the estrogen receptor α (ESR1), using transactivation assays are in the low pM range (Lussenburg et al. 2018; Miller et al. 2001). DES also affects mitochondria and microtubules in a higher µM range (Delp et al. 2019; Hallinger et al.2020; Metzler et al. 1995).		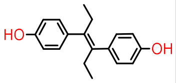
MW [g/mol]: 268.4	LogP: 5.07	SMILES: CC\C(=C(\CC)C1=CC=C(O)C=C1)C1=CC=C(O)C=C1

**MPP+**, 1-methyl-4-phenylpyridine, (Cas: 48134-75-4) is the neurotoxic metabolite of MPTP (1-methyl-4-phenyl-1,2,5,6-tetrahydropyridine). MPP+ is selectively toxic to dopaminergic neurons due to its uptake by the dopamine transporter (DAT). Toxicity of MPP+ is caused by mitochondrial complex I inhibition (Terron et al. 2018). Effects on dopaminergic neurons occur at low µM extracellular concentrations, due to strong intracellular (and intramitochondrial) accumulation. Peak concentrations in mouse brain have been reported to be 40-50 pmol/mg protein (~ 10 µM) (Schildknecht et al. 2015).		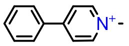
MW [g/mol]: 170.2	LogP: -0.33*	SMILES: C[N+]1=CC=C(C=C1)C1=CC=CC=C1

**Rotenone** (Cas: 83-79-4) is a plant derived rotenoid used as insecticide, piscicide and pesticide. It inhibits mitochondrial complex I (bovine heart mitochondria IC50: 16 nM, Purohit et al. 2008; HepG2 IC50: ~ 400nM, Hallinger et al. 2020). It is considered a neurotoxicant, especially for dopamine neurons (Terron et al. 2018) and it affects several neurodevelopmental key processes (e.g. neurite outgrowth – Blum et al. 2023). It has also been reported to inhibit tubulin polymerization (porcine brain tubulin 4.3 µM (Gertsch et al. 2007); goat brain tubulin 12 µM (Srivastava et al. 2007)) and mitochondrial movement in neurons (Borland et al. 2008).		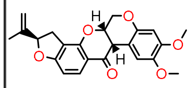
MW [g/mol]: 394.4	LogP: 4.10	SMILES: [H][C@@]12COC3=C(C=C(OC)C(OC)=C3)[C@]1([H])C(=O)C1=CC=C3O[C@H](CC3=C1O2)C(C)=C

**Valinomycin** (Cas: 2001-95-8) is a cyclic depsipeptide antibiotic from Streptomyces composed of 14 amino acids. It acts as a potassium ionophore (Rose et al 2007). Mitochondria are known to express potassium ion channels which are known to affect cell survival (Szewczyk et al. 1995). Valinomycin is expected to disturb the potassium distribution in mitochondria and to dissipate the charge gradient across the inner mitochondrial membrane (besides its effects on plasma membrane potassium ion gradients).		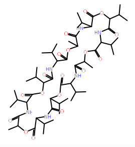
MW [g/mol]: 1111.3	LogP: 1.30*	SMILES: CC(C)C1NC(=O)C(OC(=O)C(C)OC(=O)C(NC(=O)C(OC(=O)C(NC(=O)C(C)OC(=O)C(NC(=O)C(OC(=O)C(NC(=O)C(C)OC(=O)C(NC1=O)C(C)C)C(C)C)C(C)C)C (C)C)C(C)C)C(C)C)C(C)C)C(C)C

Fig. S1
Suciu *et al.*, 2023

Supplementary Fig. S1: Mechanistic description of the 7 DNT assay hits

Compounds' chemical features as well as biological modes of action are summarized in the table.

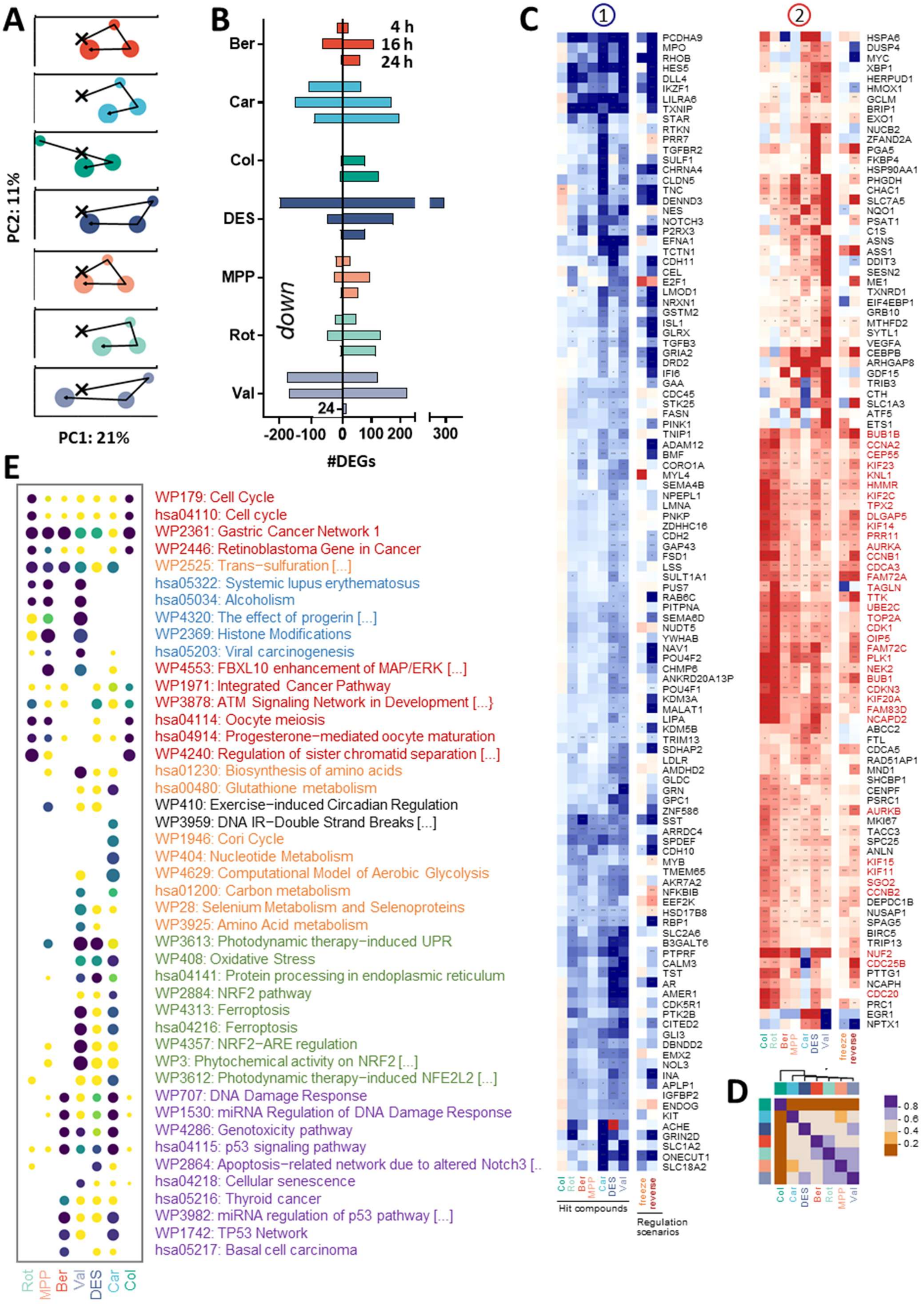

Fig. S2
Suciu *et al.*, 2023

Supplementary Fig. S2: Overview of de-regulated genes over time

Transcriptome data were obtained from LUHMES cells treated with 7 different toxicants for 4 h, 16 h and 24 h, as outlined in Fig. 1A. The following concentrations were used: 64 µM carbaryl (Car), 40 nM colchicine (Col), 94 µM berberine chloride (Ber), 21 µM diethylstilbestrol (DES), 4.4 µM rotenone (Rot), 10 µM MPP+ and 17 nM valinomycin (Val). Significant changes of mRNA levels were identified by differential gene expression analysis: for each time point, exposure conditions were compared to corresponding solvent (DMSO) controls. As output metrics, we provide the average log2 fold changes (FC) for each transcript, including the standard deviation, and the statistical significance of the change in Suppl. Table S1. **A**) Log2 FC data from all transcriptome conditions (3 times × 8 treatments) were analyzed together in a joint principal component space, with axes scaled according to the variances covered. For better overview, data for each compound are shown on separate maps. All 2D maps have the same axes and can therefore be directly compared. The dots represent the different time points connected by a black line in chronological order. The symbol "✕" marks the control condition, then 4 h, 16 h and 24 h follow. **B**) The absolute numbers of differentially expressed genes (DEGs) per exposure time are displayed. Up- and down-regulated DEGs are shown in the left- and right bar chart, respectively. **C**) The enlarged heatmaps are magnifications of regions 1+2 of the heatmap shown in Fig. Fig. 2C. Cluster 1 shows the major down-regulated consensus genes; cluster 2 shows the major up-regulated consensus genes. Note that the test system used (LUHMES) has a dynamic baseline: the cells change their differentiation state and gene expression over time also without component treatment. Each statement on gene regulation is therefore in reality a 3-point comparison (time point 1, time point 2 with solvent, time point 2 with toxicant). To allow a better interpretation of the 2-point comparison heatmaps, we added two further data columns: "freeze" shows the regulations that would have been seen, if the toxicant had stopped the differentiation process 100% at the d2 time point (start of incubation); "reverse" shows the regulations that would have been seen, if a toxicant had de-differentiated the d2 neural cells to their d0 ground state. The blue-white-red colour scale indicates fold-changes. The full blue colour is used for down-regulations ≥ 4 fold; the full red colour – for up-regulations ≥ 4 fold. Asterisks mark significant changes. **D**) The pairwise Pearson correlations between the treatment conditions were computed and displayed in a heatmap. Similarity scores are binned in 0.2 unit intervals and color-coded. **E**) The biological pathways from the Wikipathways (WP) and KEGG (hsa) databanks were analysed for overrepresentation in the transcriptome dataset shown in **A**, **B**. Pathways with an adjusted p-value < 0.05 (in any of the treatments) were displayed in a dot plot. The adjusted p-value is encoded by a purple – yellow color scale. Purple indicates the highest significance, green codes intermediate significances, yellow is used for low ones. The relative percentage of deregulated genes in a pathway is indicated by the dot size. Related pathways are highlighted by text color.

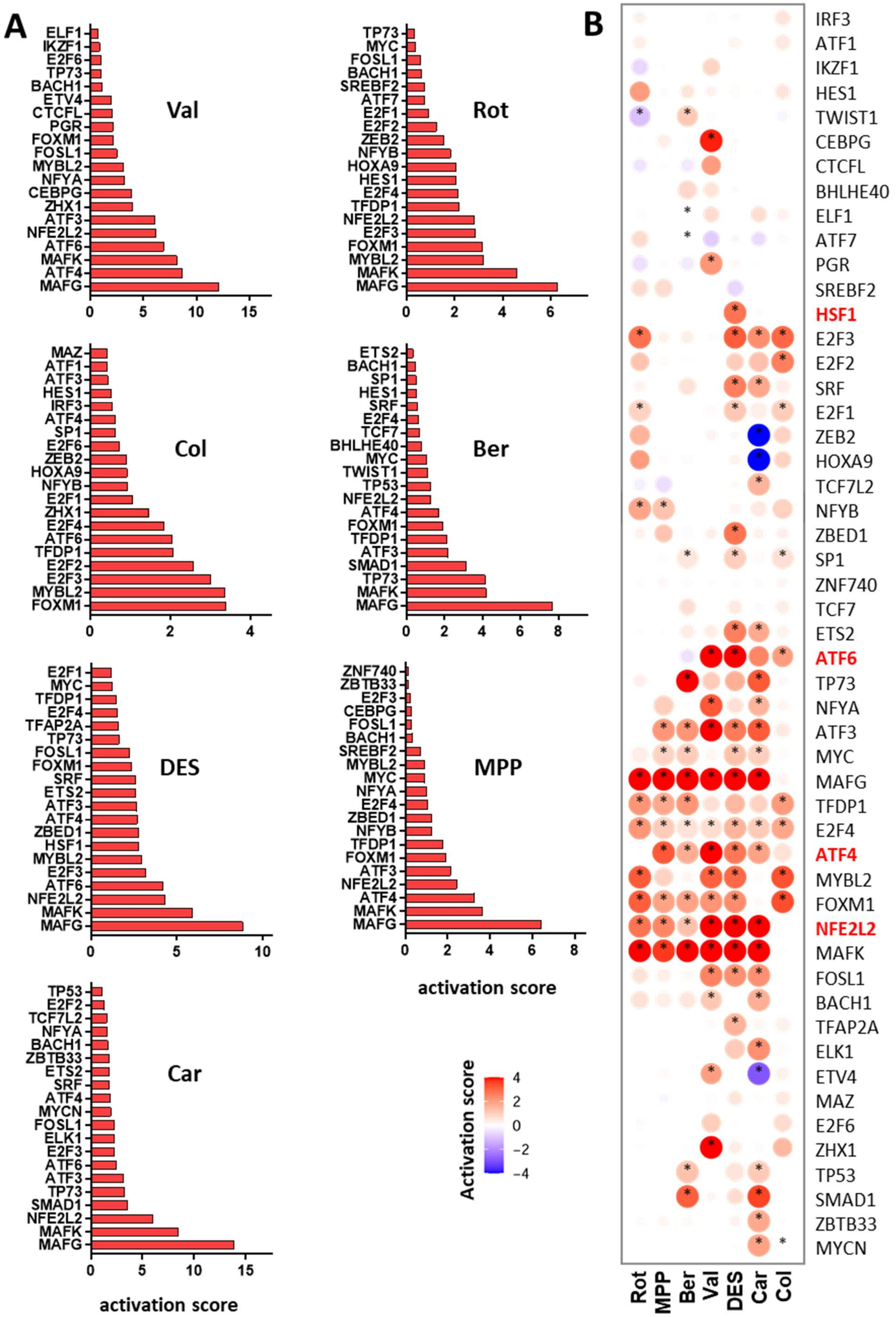

Fig. S3
Suciu *et al.*, 2023

Supplementary Fig. S3: Display of transcription factors with the highest predicted activity scores at 16 h

Differentially expressed genes were identified as in Fig. S2 (Suppl. Table S1). The pattern of activated genes was used to predict transcription factors controlling them. For this, manually curated data (DoRothEA database) on human regulons was used. Modified overrepresentation statistics were used to derive transcription factor activation scores (Garcia-Alonso et al. 2019). **A**) The top 20 up-regulated transcription factors (TF) after 16 h treatment with each of the tested neurotoxicants are ordered by their activity scores. The plots showing the top down-regulated TF are found in Fig. S5B. **B**) For comparison, data from A) are visualized side-by-side in a dot plot, where columns represent the treatment conditions and rows represent the TF. The blue-white-red color scale indicates activity scores (0.25x – 4x), while asterisks mark significant changes (p-value < 0.05). Highlighted in red are the TF whose regulation over time is plotted in Fig. 2F.

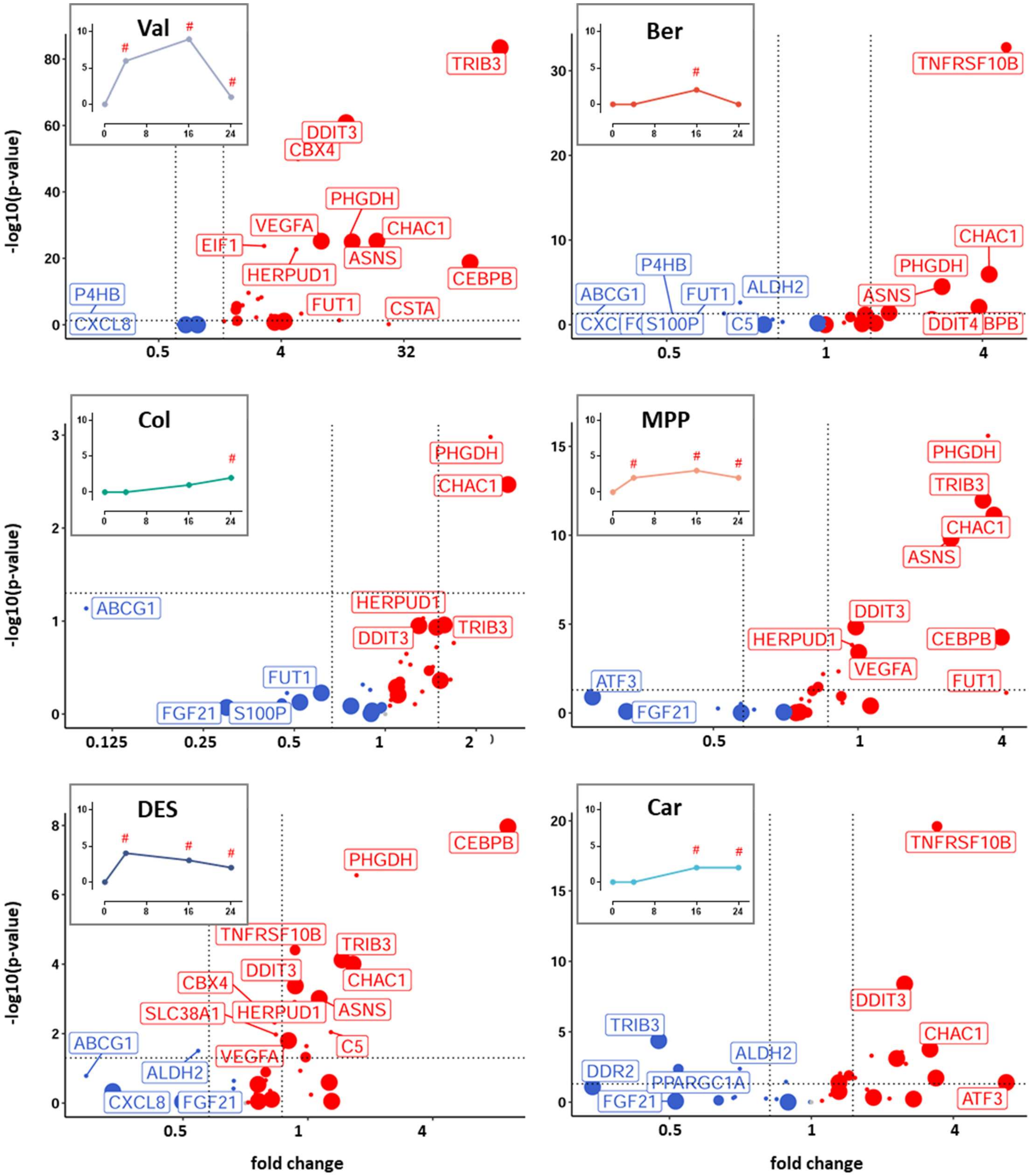

Fig. S4
Suciu *et al.*, 2023

Supplementary Fig. S4: Activity predictions for transcription factor ATF4

Differentially expressed genes were identified as in Fig. S2 (Suppl. Table S1). The volcano plots show the target genes of transcription factor ATF4 after 16 h exposure to the indicated toxicants. For each gene, the fold change values (x-axis) were plotted against their p-values (y-axis). Red colours indicate that the gene regulation has the same direction as the biological effect of ATF4 on this gene. Blue indicates that genes are regulated in opposite direction than expected, if they were controlled by ATF4. The inserts show the predicted enrichment scores for ATF4 (y-axis) over time (x-axis), from 0 until 24 h treatment. # p-value < 0.05.

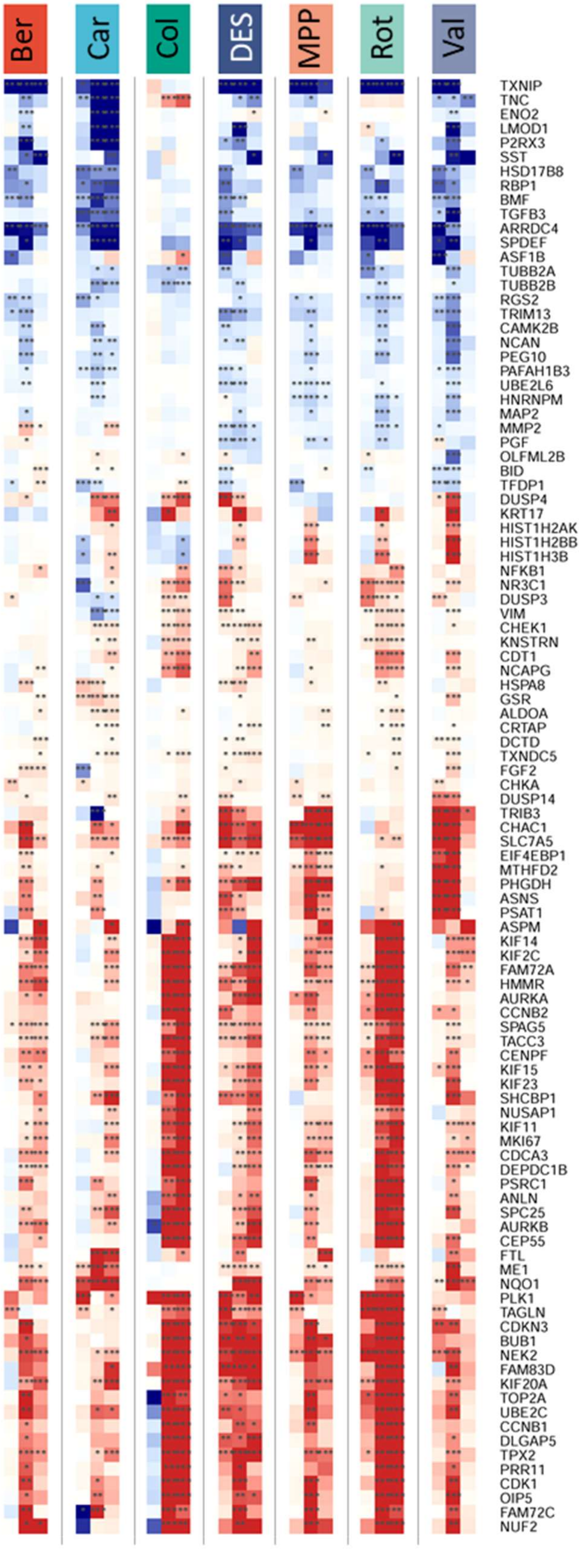

Fig. S5
Suciu *et al.*, 2023

Supplementary Fig. S5: Expression of consensus deregulated genes at 4, 16 and 24 h

Differentially expressed genes were identified as in Fig. S2 (Suppl. Table S1). The genes with an adjusted p-value ≤ 0.05 and an absolute FC > 1.5 were considered "consensus genes" and selected for further analysis. Those shared by ≥ 5 compounds (102 out of 3255 measured genes) were selected for display. The heat map gives an overview of the gene hits for all test compounds after 4, 16 and 24 h exposure time. The color scale spans from blue (4-fold down) over white (no regulation) to red (4-fold up).

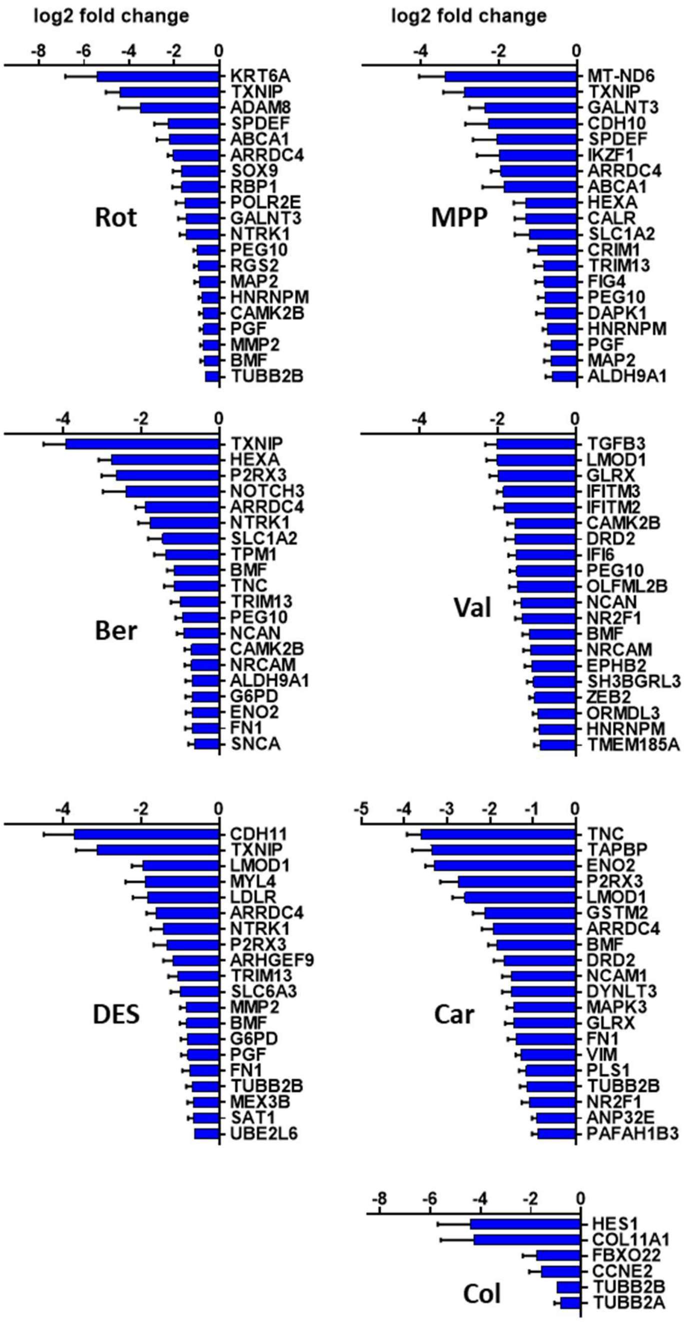

Fig. S6
Suciu *et al.*, 2023

Supplementary Fig. S6: Display of genes with highest down-regulation at 16 h

Differentially expressed genes were identified as in Fig. S2 (Suppl. Table S1). For each test compound the significantly deregulated genes (adjusted p-value <= 0.05) were ranked according to the fold change statistic. The top 20 down-regulated genes at 16 h were selected and displayed in a bar plot for each toxicant.

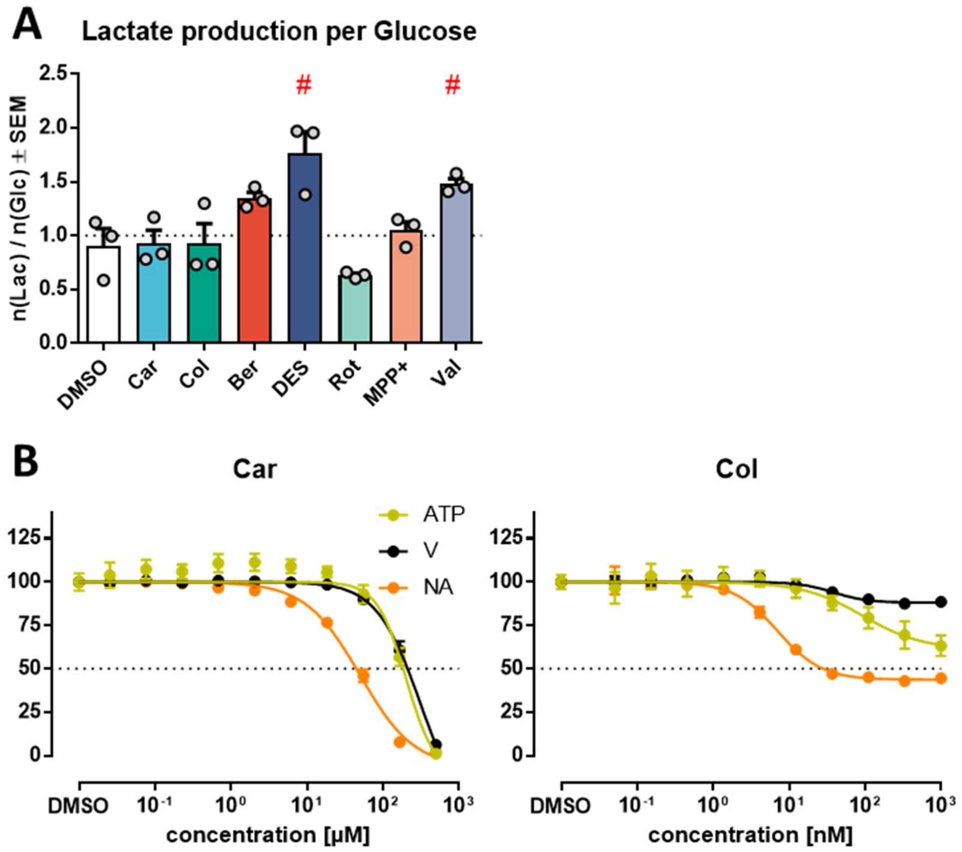

Fig. S7
Suciu *et al.*, 2023

Supplementary Fig. S7: Effect of toxicants on neuronal ATP production

Immature neurons (day 2 LUHMES) were exposed for 24 h to developmental neurotoxicants (DNT): 20 µM carbaryl (Car), 100 nM colchicine (Col), 15 µM berberine chloride (Ber), 10 µM diethylstilbestrol (DES), 1 µM rotenone (Rot), 10 µM MPP+ and 10 nM valinomycin (Val). **A**) The rate between molecules of lactate produced per molecules of glucose consumed was calculated for each compound and displayed in a bar plot. **B**) Concentration-response curves for the compounds carbaryl (Car) and colchicine (Col), which showed no activity on mitochondrial respiration (Fig 5C). Intracellular ATP was measured and plotted together with viability (V) and neurite area (NA). Data are means ± SEM of independent replicates.

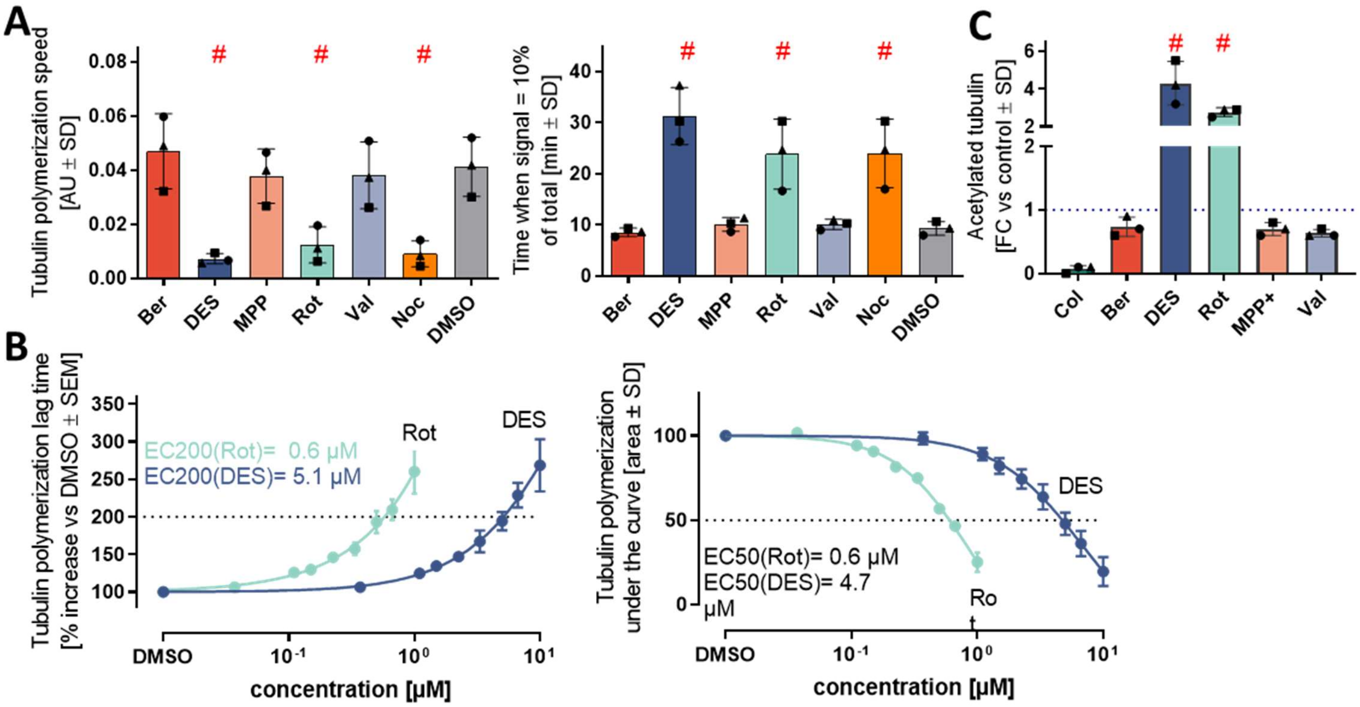

Fig. S8
Suciu *et al.*, 2023

Supplementary Fig. S8: Effect of toxicants on microtubule polymerization

Immature neurons (day 2 LUHMES) were exposed for 24 h to developmental neurotoxicants (DNT). **A**) Polymerization speed and onset derived from the *in vitro* microtubule polymerization assay using a fixed concentration (EC10V) for each test compound: 20 µM carbaryl (Car), 100 nM colchicine (Col), 15 µM berberine chloride (Ber), 10 µM diethylstilbestrol (DES), 1 µM rotenone (Rot), 10 µM MPP+ and 10 nM valinomycin (Val). 1 µM nocodazole (Noc) served as positive control. The negative control treatment contained 0.5 % DMSO. Data are means of 3 independent replicates ± standard deviation (SD). **B**) Area under the curve (AUC) and polymerization speed were quantified for the 2 active compounds (Rot, DES) at different concentrations. **C**) Bar plot showing protein levels for acetylated Tubulin (Lys40). Data in figures A), C) are mean ± SD and statistical differences between the treatments and the control were evaluated by an analysis of variance (ANOVA) followed by Dunnett's post hoc test ($p < 0.05$, indicated by #). Individual samples are represented as dots; dot symbol indicates paired replicates.

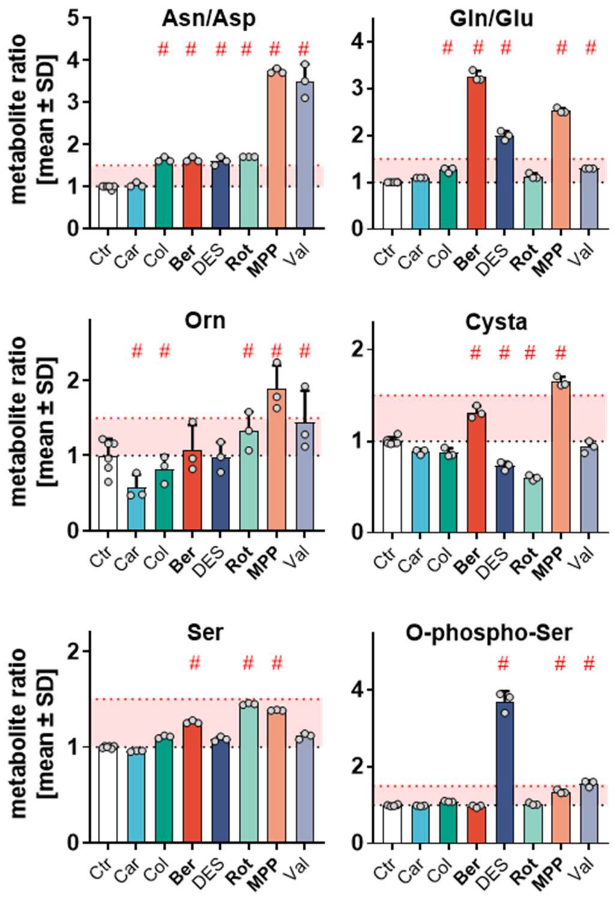

Fig. S9
Suciu *et al.*, 2023

Supplementary Fig. S9: Intracellular amino acid changes in immature neurons exposed to neurodevelopmental toxicants

Bar plots of exemplary metabolites and metabolite ratios for all seven toxicants are displayed. Data are means ± SD from at least three independent experiments. # adjusted p-value < 0.05; Cysta, cystathionine; L-Orn, ornithine.

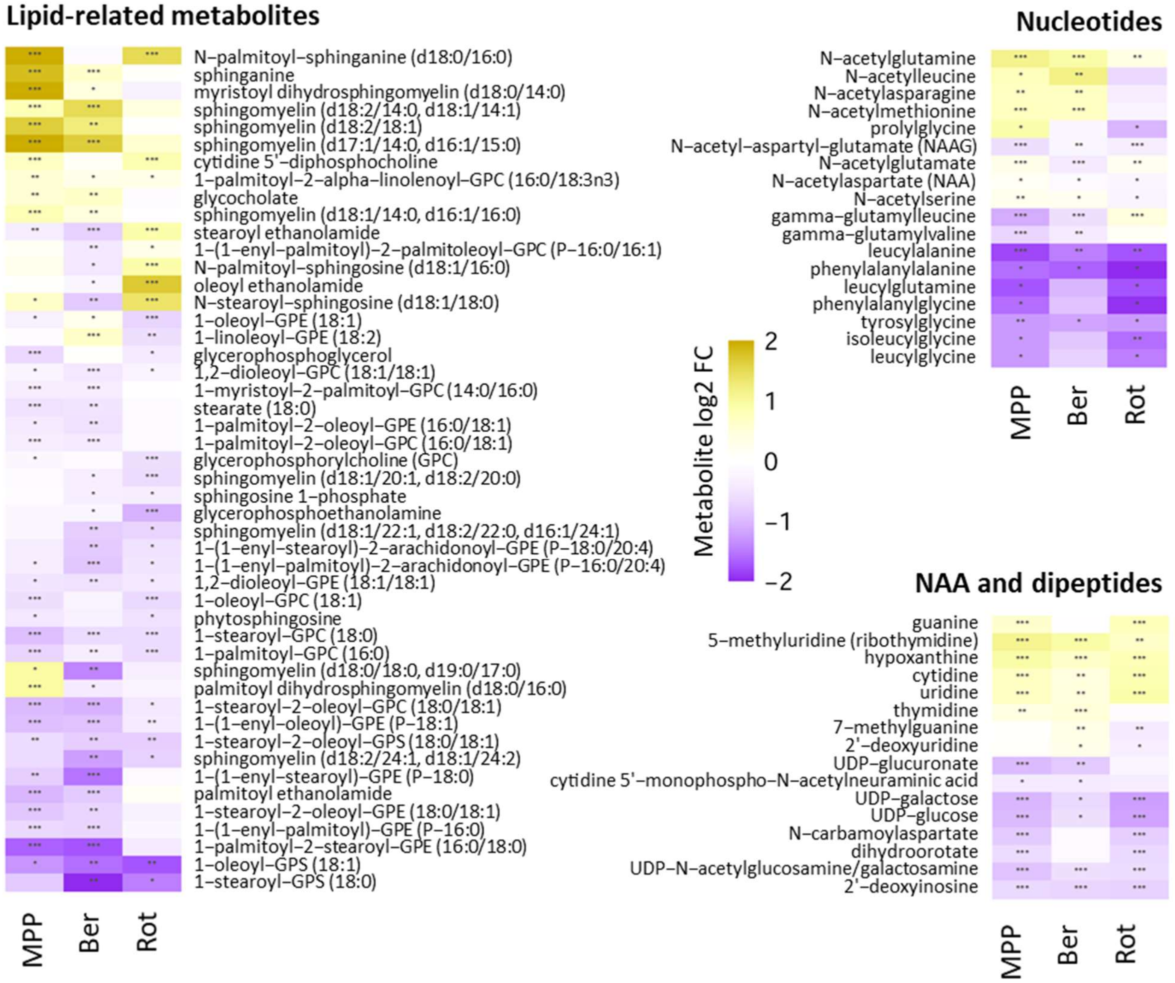

Fig. S10
Suciu *et al.*, 2023

Supplementary Fig. S10: Other metabolic changes induced by c-I inhibitors in neurons

LUHMES cells (d2) were exposed to 12.5 µM berberine (Ber), 10 µM MPP+ (MPP) or 0.5 µM rotenone (Rot) for 24 h. Intracellular metabolite concentrations were determined by LC-MS. Significant metabolite changes (vs solvent control) were identified for each treatment condition. The heatmap displays all metabolites changed significantly by at least 2 toxicants which fall under the categories lipids, neucleotides, N-acetylated amino acids (NAA) and dipeptides. A linear yellow-white-purple color scale indicates fold-changes (0.25x – 4x) and asterisks mark significant changes. Dendrogram not shown. * adjusted p-value ≤ 0.05; ** adjusted p-value ≤ 0.01; *** adjusted p-value ≤ 0.001; DEG, differentially expressed gene. GPC, glycerophosphoryl-choline; GPE, glycerophosphoryl-ethanolamine; GPI, glycosylphosphatidyl-inositol; GPS, glycerophosphoryl-serine; NAA, N-acetyl amino acids.

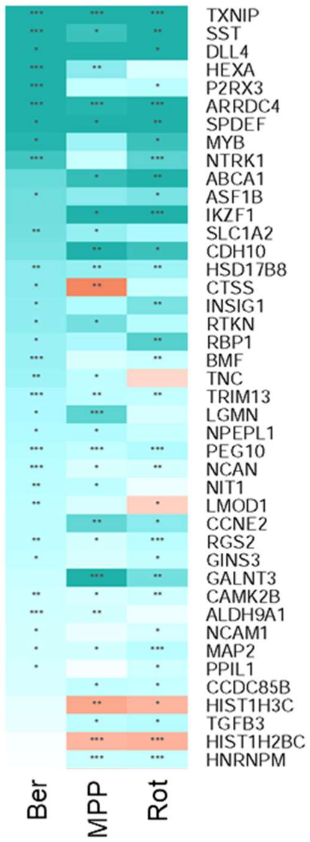

Fig. S11
Suciu *et al.*, 2023

Supplementary Fig. S10: Consensus transcriptome changes induced by c-I inhibitors in neurons

Transcriptome data were obtained from LUHMES cells treated with berberine (Ber), rotenone (Rot) and MPP (MPP+) for 4 h, 16 h and 24 h, as outlined in Fig. S2. Differentially expressed genes were identified (see full details in Suppl. Table S1). For each compound and gene, the expression change was followed over time, and only the peak data (defined by significance) were kept as a dimensionality-reduced data set. Next, the genes with an adjusted p-value ≤ 0.05 and an absolute fold change (FC) > 1.5 (differentially expressed in ≥ 2 out of the 3 toxicant treatments) were selected for further analysis. The down-regulated “consensus genes” (sorted in ascending order of the Ber FC values) are visualized in a heatmap. The color scale spans from blue (4-fold down) over white (no regulation) to red (4-fold up).

	Fold change				
**Metabolite**	**Ber**	**MPP**	**Rot**	**Disease**	**Reference**
saccharopine	**297**	**44**	**112**	saccharopinuria	Carson et al. 1968
α-aminoadipate	**5.9**	**12**	**10**	2-aminoadipic aciduria	Danhauser et al. 2012
glutarate	**5.8**	**11**	**6.8**	glutaric aciduria	Hoffmann and Zschocke 1999
KIC	**3.0**	**3.3**	**2.2**	maple syrup urine disease	Strauss et al. 1993
KMV	**5.1**	**5.0**	**3.5**		
KIV	**4.5**	**4.7**	**3.2**		
α-KGM	1.3	**1.5**	1.0	hepatic encephalopathy	Vergara et al. 1974
2-OH-glut	**1.6**	**1.5**	**2.1**	2-hydroyglutaric aciduria	Du and Hu 2021
OH-phe-Pyr	**1.8**	**1.7**	1.1	tyrosinemia type III	Ruetschi et al. 2000
OH-phe-Lac	**3.0**	**2.8**	**2.0**		
EMA	**1.5**	**3.3**	**1.6**	ethylmalonic encephalopathy	Olivieri et al. 2021
5-oxo-Pro	1.3	1.4	0.9	pyroglutamic aciduria	Shi et al. 1996

Fig. S12
Suciu *et al.*, 2023

Supplementary Fig. S12: Metabolites linked to aminoacidopathies

Metabolome data were obtained from LUHMES cells treated with berberine (Ber), rotenone (Rot) and MPP (MPP+) for 24 h, as outlined in Fig. S9. Differentially abundant metabolites were identified (see full details in Suppl. Table S1). For metabolites reported to accumulate in aminoacidopathies, we extracted the fold change statistic and displayed it for each of the tested mitotoxicants. Red labelling indicates a significant cellular accumulation (adjusted $p < 0.05$); all metabolites that changed > 1.5 fold are marked by "bold" formatting. α-KMG, α-ketoglutaramate; EMA, ethylmalonate; KIC, α-keto-Leu (keto-isocaproate); KIV, α-keto-Val (keto-isovalerate); KMV, α-keto-Ile (keto-methylvalerate); 2-OH-glut, 2-hydroxyglutarate; OH-phe-Lac, 3-(4-hydroxyphenyl)lactate; OH-phe-Pyr, 4-hydroxyphenylpyruvate; 5-oxo-Pro, 5-oxo-proline.

A

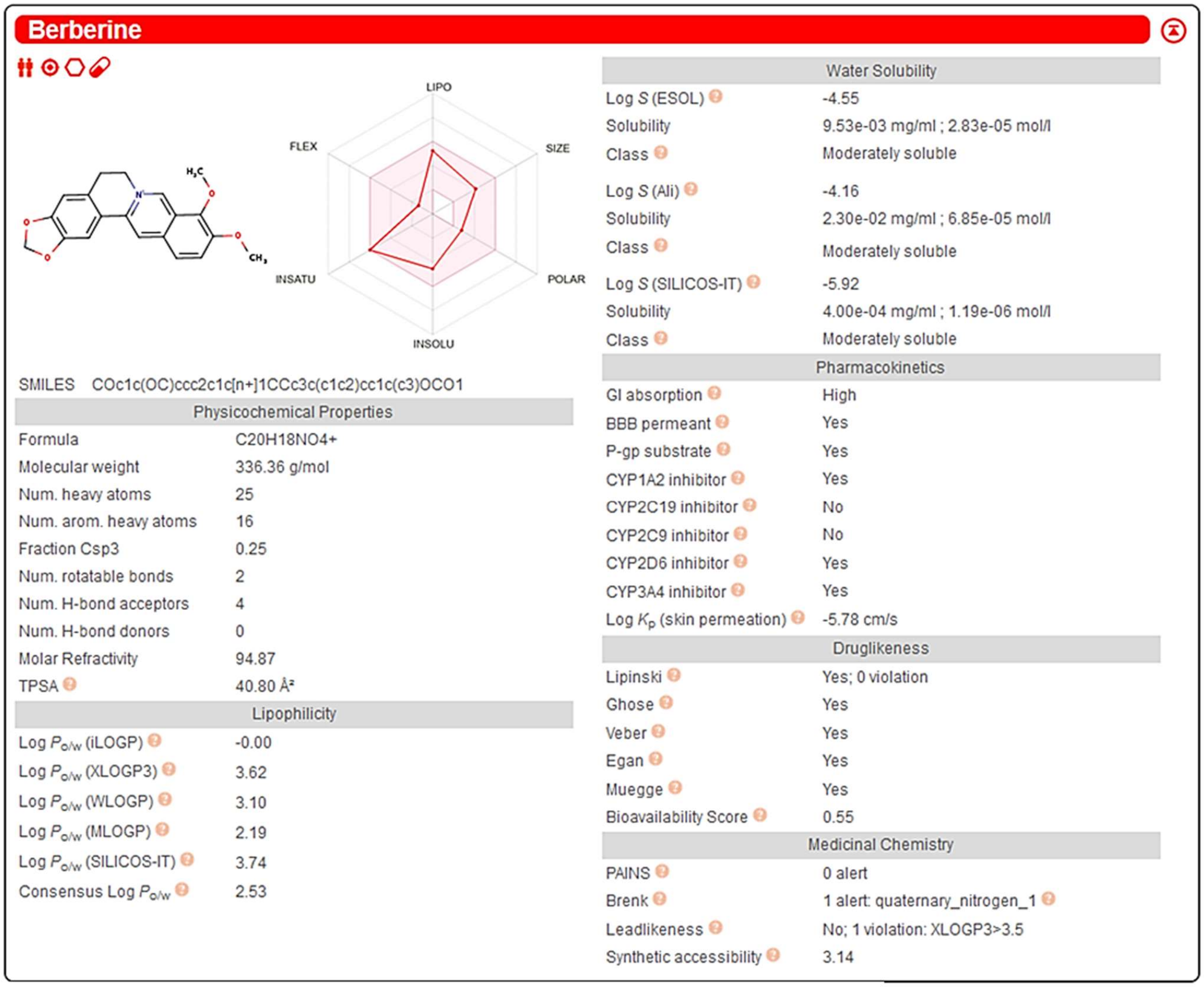

SMILES COc1c(OC)ccc2c1c[n+]1CCc3c(c1c2)cc1c(c3)OCO1

Physicochemical Properties	
Formula	C20H18NO4+
Molecular weight	336.36 g/mol
Num. heavy atoms	25
Num. arom. heavy atoms	16
Fraction Csp3	0.25
Num. rotatable bonds	2
Num. H-bond acceptors	4
Num. H-bond donors	0
Molar Refractivity	94.87
TPSA	40.80 Å²

Lipophilicity	
Log $P_{o/w}$ (iLOGP)	-0.00
Log $P_{o/w}$ (XLOGP3)	3.62
Log $P_{o/w}$ (WLOGP)	3.10
Log $P_{o/w}$ (MLOGP)	2.19
Log $P_{o/w}$ (SILICOS-IT)	3.74
Consensus Log $P_{o/w}$	2.53

Water Solubility	
Log *S* (ESOL)	-4.55
Solubility	9.53e-03 mg/ml ; 2.83e-05 mol/l
Class	Moderately soluble
Log *S* (Ali)	-4.16
Solubility	2.30e-02 mg/ml ; 6.85e-05 mol/l
Class	Moderately soluble
Log *S* (SILICOS-IT)	-5.92
Solubility	4.00e-04 mg/ml ; 1.19e-06 mol/l
Class	Moderately soluble

Pharmacokinetics	
GI absorption	High
BBB permeant	Yes
P-gp substrate	Yes
CYP1A2 inhibitor	Yes
CYP2C19 inhibitor	No
CYP2C9 inhibitor	No
CYP2D6 inhibitor	Yes
CYP3A4 inhibitor	Yes
Log K_p (skin permeation)	-5.78 cm/s

Druglikeness	
Lipinski	Yes; 0 violation
Ghose	Yes
Veber	Yes
Egan	Yes
Muegge	Yes
Bioavailability Score	0.55

Medicinal Chemistry	
PAINS	0 alert
Brenk	1 alert: quaternary_nitrogen_1
Leadlikeness	No; 1 violation: XLOGP3>3.5
Synthetic accessibility	3.14

B

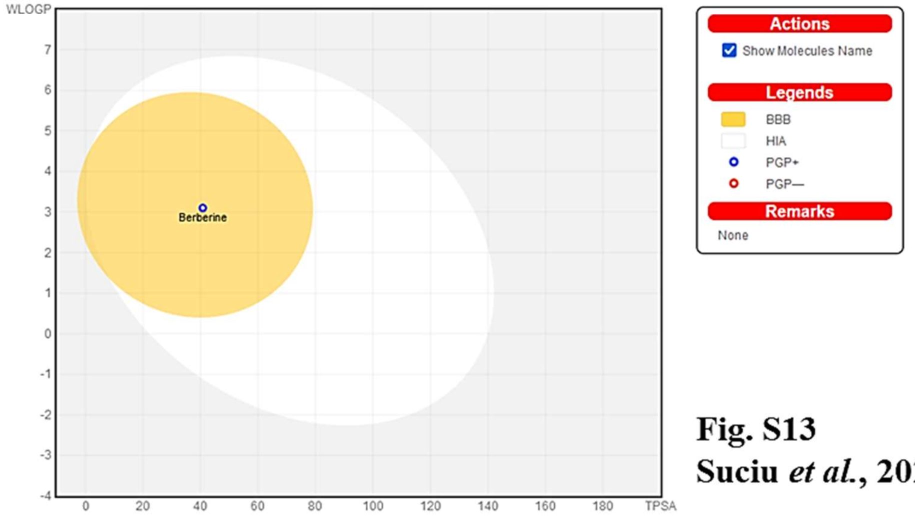

Fig. S13
Suciu *et al.*, 2023

Supplementary Fig S13: Pharmacokinetic parameters of berberine predicted by SwissADME

Panel **A**) portrays the pharmacokinetic characteristics of berberine, as predicted by the SwissADME tool (Daina et al. 2017). Molecular weight, lipophilicity (logP), aqueous solubility, and other descriptors are listed, offering perspectives on the compound's potential bioavailability and drug-likeness. In Panel **B**), the Brain Or IntestinaL EstimateD permeation method (BOILED-Egg) is employed to visualize the anticipated permeation of berberine within brain and intestinal tissues.

Supplementary Results

Suppl. results 1: Detailed pattern of transcriptome changes induced by toxicants over time

Data on transcriptome changes were obtained for three exposure times. First, all transcriptome data (times × compounds) (Fig. 2A) were analyzed together in a joint two-dimensional (2D) principal component analysis (PCA) space (Fig. 2B, S2A). Already after 4 h, strong changes were evident, and the 16 h time point separated clearly from 4 h and 0 h. The 24 h time point separated from 4 h and 16 h, but was close to the 0 h condition. This suggests that the altered transcriptome starts to return towards a baseline after 24 h prolonged exposure. Interestingly, most compounds showed similar time-tracks on a 2D PCA map. This indicates a large degree of similarity in the transcriptome responses; only Col triggered a somewhat different pattern (Fig. S2A).

In a next step, we were interested in a comparison of toxicant-responses on a gene-by-gene level. We reduced the 21 exposure situations (3 times × 7 compounds) to 7 conditions, by always picking the most significant gene expression change for each gene and compound from the time course data. This allowed a cross-comparison, also for compounds that triggered expression changes with different peak times. All genes that were changed by at least two compounds were selected for a display. By analyzing changes across compounds, two clusters of similarly-behaving genes emerged (Fig. 2C). The significantly up-regulated cluster encompassed 2 main functional categories: stress response markers (e.g. *NQO1*, *CHAC1*) and cell cycle regulators (cyclins *CCNB1*, *CCNB2*; kinases *PLK1* and *AURKA*; components of the spindle assembly checkpoint *BUB1* and *BUB1B*; kinesins *KIF20A*, *KIF23*, *KIF11*, *KIF15*) (Fig. S2C). Car, DES and Val had the most pronounced effect on the transcriptome (Fig. 2D, S2E). The group of mitotoxicants (Ber, Rot, MPP and Val) triggered closely related transcriptome changes (Fig. S2D). In the case of Ber, MPP, Car, DES and Val, most of the seemingly upregulated cell cycle markers indicated an impaired differentiation, i.e. a failure of the cell cycle exit taking normally place in the cultures at this differentiation stage. Rot and Col had particularly pronounced effects on cell cycle genes, i.e. they partially reversed the differentiation.

Another analytical approach profiled the top 10% genes upregulated by each compound. Upregulated stress response genes were abundant in this group (Fig. 2E, F). Expression of NRF2 targets (*NQO1*, *ME1*) was elevated by all treatments, except Col. Genes under the control of ATF4 (*CHAC1*, *ASNS*, *MTHFD2*) were induced by MPP, Ber, Val, DES and Car. DES uniquely enhanced the expression levels of HSF1-regulated genes (*HSPB1*, *HSPA1B*). At 16 h, the activity of ATF4 and NRF2 was predicted to be significantly increased by most toxicants (Fig. S3), whereas the time dependency was different for each treatment (Fig. 2F, S4). Analysis of overrepresented pathways indicated a change of transsulfuration (Fig. S2E). This is a typical outcome of ATF4/NRF2 activation and confirms a general stress response.

To gain an understanding of the timing of transcriptome changes, we plotted the consensus genes (regulated by ≥ 5 compounds) responses across all time points. Concerning this group of transcripts, Ber, MPP, Rot and Val had their peak at 16 h, whereas the other compounds triggered maximal effects at 24 h (Fig. S5). We mainly focused here on descriptions of positive regulations, since they resulted in biologically coordinated responses, evident as pathway activations (Fig. S2E). Notably, some responses, e.g. of Ber or Val were transient, i.e. the consensus genes regulated strongly at 16 h were often returnig toward their basal expression at 24 h despite the continued presence of the toxicants.

The down-regulated genes gave a less coherent picture, and no pathways were clearly overrepresented. However, when hits of the most regulated transcripts were assembled for each compound, they contained many factors associated with the phenotype of the neural precursor cells and neurons (Fig. S6). Examples are *P2RX2*, *NTRK1* and *NOTCH3*. Also conspicuous were *TXNIP* and *HEXA*, both associated with neurodegeneration and accelerated aging.

Suppl. results 2: Changes in amino acid metabolism determined by a targeted approach

The similarity of effects may be assessed in the future by extensive metabolomics studies. For instance, a panel of 700 metabolites has been suggested to be explored for use in regulatory settings. However, the resource demands for such approaches are very high, and there are few metabolites that are consistently detected and quantified across various modern platforms (Sostare et al. 2022). In most studies, the majority of altered metabolites are amino acids, and there is a well-established, traditional method (successfully in use for decades) for a robust quantification of this subset of metabolites: HPLC-based post-column-derivation amino acid analysis (Ersser and Davey 1991). Using this approach, we checked which of the cellular amino acid concentrations was affected by the toxicants. The response patterns of mitochondrial toxicants (MPP, Ber, Rot and Val) were indeed largely similar (Fig. 6). For instance, ornithine (Orn), asparagine (Asn), glutamine (Gln) and serine (Ser) were increased, while aspartate (Asp) and glutamate (Glu) were depleted.

One interpretation, consistent with these findings, is that the cells' nitrogen balance was altered, e.g. by use of amino acids as energy substrate. An alternative hypothesis is that the cells lacked amino acid precursor normally generated by the TCA in mitochondria (oxaloacetate and 2-oxoglutarate). These two metabolites are normally used in the synthesis of aspartate and glutamate. The two changes hypothesized above may also occur concurrently.

When we calculated the Asn/Asp ratio, we found that all toxicants (besides Car) increased it by at least 1.5-fold (Fig. S9). The change was significant for all mitochondrial toxicants. The Gln/Glu ratio was increased by the mitochondrial toxicants Ber, Val and MPP, but not by Rot (Fig. 6, S9). This may indicate some differences in the toxicity pathways. Alternatively, it may be explained by the concentration and time selection for this experiment. While we obtained clear evidence of some co-ordinated metabolite changes, the elucidation of reasons for differences and inconsistencies will require extensive further work. For instance, metabolome and transcriptome changes will need to be integrated. One example for this is that Asp and Glu can be converted directly to Asn and Gln by asparagine synthetase (ASNS) (Balasubramanian et al. 2013). We found the gene expression of this metabolic enzyme to be increased by all hits except Rot and Col (Fig. 2E).

Another couple of amino acids that is metabolically coupled is Pro and Orn. Orn accumulated in the presence of Rot, MPP and Val, while the toxicants Car and Col depleted it (Fig 6, S9). Orn is a precursor for the synthesis of Pro, but it may take several other functions (nitrogen sink and polyamine synthesis). Pro may be increased by mitochondrial toxicants in some cells (it is generated under reductive stress (excessive supply of NADH) from Glu). However, there have also been reports that Pro can be used up to maintain some mitochondrial function upon c-I inhibition (Pallag et al. 2022). The decrease of Pro in the presence of Ber, MPP and Val supports the latter finding.

The tripeptide glutathione (GSH, γ-Glu-Cys-Gly) was depleted by all c-I inhibitors (Fig 6). This is not surprising, since GSH is known to play a role in reducing the reactive oxygen species (ROS) formed upon inhibition of mitochondrial ETC (Espinosa-Diez et al. 2015; Zorov et al. 2014). Cystathionine (Cysta), a Cys precursor, was increased by Ber and MPP, but decreased by DES and Rot (at 24 h) (Fig S9). Upon oxidative stress, GSH and its precursors were shown previously to follow a 2-step time-dependent response: after an early drop, the cellular counter-regulation (orchestrated by NRF2/ATF4) led in many cases to an accumulation that was higher than control levels (Suciu et al. 2023; Wijaya et al. 2022). For the different inhibitors, the speed of GSH re-accumulation might be different, which would explain the apparently inconsistent regulation of Cysta at 24 h. Detailed time-course studies will be required for further clarification.

The final two metabolites that we selected here to exemplify differential responses are serine (Ser) and phospho-Ser. They are related to the glycolysis intermediate 3-phosphoglycerate (3-PG) and not directly affected by mitochondrial metabolism. The c-I inhibitors Ber, Rot and MPP increased Ser levels. This may be an indication of the largely increased glycolysis in cells treated with these compounds (Fig. 4A, B). A contrasting molecular response was e.g. seen for DES, which strongly increased phospho-Ser, but not Ser.

In summary, the data shown here point into two directions. On the one hand, c-I inhibitor showed a pattern of metabolic changes that was largely similar and that agreed (post-hoc) with biochemical expectations. On the other hand, there was also clear heterogeneity between mechanistically related compounds, and there was some overlap of metabolic changes with mechanistically-unrelated compounds. We conclude from this, that single metabolites have little toxicological information value. Moreover, even a group of two-three dozen metabolites in unlikely to indicate a mode of action or to lead to a sharp distinction of related and unrelated compounds. The latter situation may be improved by the study of a time-concentration matrix for each compound and/or by a meticulous selection of metabolites with high separation potential. Another strategy may be to look at a broader panel of metabolites, using a modern, more sensitive and less biased (untargeted) metabolomics approach. This latter strategy was explored below.

Suppl. results 3: Methodological considerations and detailed rationale of metabolic changes related to the TCA cycle perturbation

The unbiased overrepresentation analysis has suggested the TCA to be amongst the main metabolic pathways deregulated by c-I inhibitors. A more biologically-oriented approach takes into account the connections of metabolites in known metabolic reactions and the direction of their regulation. One powerful approach for this is metabolic flux analysis. This has been used repeatedly to demonstrate that the TCA cycle is slowed down upon c-I inhibition and that several of its entry and exit reactions may change (Buescher et al. 2015; Krug et al. 2014; Lorendeau et al. 2017; Mullen et al. 2011; Pachnis et al. 2022; Sullivan et al. 2015). The technology requires the use of isotope-labelled metabolic precursors and of complex mathematical models for data processing.

We used knowledge of entry pathways to obtain more detailed information:

The canonical fueling reaction of the TCA is the generation of citrate from oxaloacetate (OAA) and acetyl coenzyme A (Ac-CoA). The latter is generated from energy substrates such as glucose, fatty acids or various amino acids. The reaction cannot take place, when OAA is depleted as a consequence of a down-regulated TCA. In this situation, the second reaction product (besides citrate), i.e. CoA cannot be formed as well. Indeed, we observed a depletion of free CoA to < 50% of control values, when cells were exposed to c-I inhibitors.

Another consequence of the down-regulated entry of Ac-CoA to the TCA is the accumulation of acetyl groups in mitochondria. This is known to lead to a transfer to mitochondrial carnitine as alternative substrate to form Ac-carnitine. The latter is typically exported to the cytosol and thus relieves the acetyl-overload of mitochondria. In confirmation of this metabolic response, we found that all c-I inhibitors caused a massive accumulation of Ac-carnitine (300%). Moreover, we found an upregulation of several acetylated AA (Fig. S10). Transfer of Ac from Ac-carnitine to AA is a known reaction to recycle carnitine for further use of mitochondrial shuttling. Both CoA depletion and Ac-carnitine accumulation are consistent with measured changes of these metabolites in the mitochondrial matrix of piericidin-treated cells in other studies (Chen et al. 2016).

When Ac-CoA is provided by glycolysis, block of the TCA has several consequences on upstream metabolites On the one hand, glycolytic flux may be increased to generate ATP independent of mitochondrial function. On the other hand, the pyruvate and the NADH generated in glycolysis is not utilized in mitochondria. This leads to an accumulation of NADH in the cell (here up to 2000% were observed) and to the reaction of excessive pyruvate and NADH to lactate (all c-I inhibitors caused a strong increase). All these changes, observed and described here, are fully aligned with a reduced TCA flux as common cause. However, it needs to be noted that NADH overflow may not only be due to glycolysis, but some residual TCA activity may also produce NADH. The overall NADH pool size would be affected by residual ETC activity (NADH is oxidized by the ETC).

Suppl. results 4: Established and novel markers of oxidative stress due to altered AA metabolism

Thiol-containing AA are nearly always affected by oxidative stress, in part by their use to generate and to replenish the redox buffer glutathione, but also as part of the counter-regulation to oxidative stress (Gutbier et al. 2018; Krug et al. 2014; Wijaya et al. 2022). Here, indeed Met and Cys levels were augmented (at the time point examined). The Met degradation product ethylmalonate (EMA) (McGowan et al. 2004) was among the consensus c-I up-regulated metabolites (Fig. 7D). Its high concentration in urine is a marker of ethylmalonic encephalopathy (Olivieri et al. 2021) and of short-chain acyl-CoA dehydrogenase deficiency (Fig. S12). The upregulated Met oxidation product methionine sulfoxide (MetSO) is a widely used marker of oxidative stress, either as small metabolite, or as part of the polypeptide chain of proteins (Gerding et al. 2019).

The most intriguing finding was the severe reduction (down to less than 10%) in N-formyl-Met (fMet) (Fig. 7D). This modified amino acid plays a unique role in translation initiation of proteins encoded by the mitochondrial DNA (mtDNA, corresponding to the AUG codon) and by other prokaryotic genomes (Lee et al. 2022; Marcker and Sanger 1964; Smith and Marcker 1968; Waller 1963). Furthermore, there is evidence of an association between a hereditary mtDNA mutation in the complex I subunit MT-ND1 and a reduction in circulating N-formyl-Met (Cai et al. 2021). The reduced fMet levels may make mitochondrial protein synthesis impossible, and this would contribute to a perturbed homeostasis of mitochondrial proteins upon prolonged toxicant exposure. The finding is particularly significant in the light of our data that non-modified Met as such was not reduced, and thus available within the cells.

Another oxidative stress marker, 5-oxo-Pro was also augmented (Fig. 7D, 9A). This was consistent with a depletion of GSH (down to less than 25%) and of its precursors γ-Glu-Gly (down to ~ 15%) and Cys-Gly (Fig. 9A). High levels of 5-oxo-Pro in urine are a marker for the diagnosis of pyroglutamic aciduria (Fig. S12), a condition associated with damage to the central nervous system (Shi et al. 1996).

References

Balasubramanian MN, Butterworth EA, Kilberg MS (2013) Asparagine synthetase: regulation by cell stress and involvement in tumor biology. Am J Physiol Endocrinol Metab 304(8):E789-99 doi:10.1152/ajpendo.00015.2013

Blum J, Masjosthusmann S, Bartmann K, et al. (2022) Establishment of a human cell-based in vitro battery to assess developmental neurotoxicity hazard of chemicals. Chemosphere 311(Pt 2):137035 doi:10.1016/j.chemosphere.2022.137035

Borland MK, Trimmer PA, Rubinstein JD, et al. (2008) Chronic, low-dose rotenone reproduces Lewy neurites found in early stages of Parkinson's disease, reduces mitochondrial movement and slowly kills differentiated SH-SY5Y neural cells. Mol Neurodegener 3:21 doi:10.1186/1750-1326-3-21

Buescher JM, Antoniewicz MR, Boros LG, et al. (2015) A roadmap for interpreting (13)C metabolite labeling patterns from cells. Curr Opin Biotechnol 34:189-201 doi:10.1016/j.copbio.2015.02.003

Cai N, Gomez-Duran A, Yonova-Doing E, et al. (2021) Mitochondrial DNA variants modulate N-formylmethionine, proteostasis and risk of late-onset human diseases. Nat Med 27(9):1564-1575 doi:10.1038/s41591-021-01441-3

Carson NA, Scally BG, Neill DW, Carre LJ (1968) Saccharopinuria: a new inborn error of lysine metabolism. Nature 218(5142):679 doi:10.1038/218679a0

Chen WW, Freinkman E, Wang T, Birsoy K, Sabatini DM (2016) Absolute Quantification of Matrix Metabolites Reveals the Dynamics of Mitochondrial Metabolism. Cell 166(5):1324-1337 e11 doi:10.1016/j.cell.2016.07.040

Daina A, Michielin O, Zoete V. SwissADME: a free web tool to evaluate pharmacokinetics, drug-likeness and medicinal chemistry friendliness of small molecules. Sci. Rep. 2017; 7:42717. doi: 10.1038/srep42717

Danhauser K, Sauer SW, Haack TB, et al. (2012) DHTKD1 mutations cause 2-aminoadipic and 2-oxoadipic aciduria. Am J Hum Genet 91(6):1082-7 doi:10.1016/j.ajhg.2012.10.006

Delp J, Funke M, Rudolf F, et al. (2019) Development of a neurotoxicity assay that is tuned to detect mitochondrial toxicants. Arch Toxicol 93(6):1585-1608 doi:10.1007/s00204-019-02473-y

Du X, Hu H (2021) The Roles of 2-Hydroxyglutarate. Front Cell Dev Biol 9:651317 doi:10.3389/fcell.2021.651317

El-Naggar AM, Eissa IH, Belal A, El-Sayed AA (2020) Design, eco-friendly synthesis, molecular modeling and anticancer evaluation of thiazol-5(4H)-ones as potential tubulin polymerization inhibitors targeting the colchicine binding site. RSC Adv 10(5):2791-2811 doi:10.1039/c9ra10094f

Ersser RS, Davey JF (1991) Liquid chromatographic analysis of amino acids in physiological fluids: recent advances. Med Lab Sci 48(1):59-71

Espinosa-Diez C, Miguel V, Mennerich D, et al. (2015) Antioxidant responses and cellular adjustments to oxidative stress. Redox Biol 6:183-197 doi:10.1016/j.redox.2015.07.008

Garcia-Alonso L, Holland CH, Ibrahim MM, Turei D, Saez-Rodriguez J (2019) Benchmark and integration of resources for the estimation of human transcription factor activities. Genome Res 29(8):1363-1375 doi:10.1101/gr.240663.118

Gerding HR, Karreman C, Daiber A, et al. (2019) Reductive modification of genetically encoded 3-nitrotyrosine sites in alpha synuclein expressed in E.coli. Redox Biol 26:101251 doi:10.1016/j.redox.2019.101251

Gutbier S, Spreng AS, Delp J, et al. (2018) Prevention of neuronal apoptosis by astrocytes through thiol-mediated stress response modulation and accelerated recovery from proteotoxic stress. Cell Death Differ 25(12):2101-2117 doi:10.1038/s41418-018-0229-x

Hallinger DR, Lindsay HB, Paul Friedman K, Suarez DA, Simmons SO (2020) Respirometric Screening and Characterization of Mitochondrial Toxicants Within the ToxCast Phase I and II Chemical Libraries. Toxicol Sci 176(1):175-192 doi:10.1093/toxsci/kfaa059

Henley DV, Korach KS (2010) Physiological effects and mechanisms of action of endocrine disrupting chemicals that alter estrogen signaling. Hormones (Athens) 9(3):191-205 doi:10.14310/horm.2002.1270

Hoffmann GF, Zschocke J (1999) Glutaric aciduria type I: from clinical, biochemical and molecular diversity to successful therapy. J Inherit Metab Dis 22(4):381-91 doi:10.1023/a:1005543904484

Krug AK, Gutbier S, Zhao L, et al. (2014) Transcriptional and metabolic adaptation of human neurons to the mitochondrial toxicant MPP(+). Cell Death Dis 5:e1222 doi:10.1038/cddis.2014.166

Lee CS, Kim D, Hwang CS (2022) Where Does N-Formylmethionine Come from? What for? Where Is It Going? What is the origin of N-formylmethionine in eukaryotic cells? Mol Cells 45(3):109-111 doi:10.14348/molcells.2021.5040

Leist M, Single B, Kunstle G, Volbracht C, Hentze H, Nicotera P (1997) Apoptosis in the absence of poly-(ADP-ribose) polymerase. Biochem Biophys Res Commun 233(2):518-22 doi:10.1006/bbrc.1997.6491

Li S, Zhao J, Huang R, et al. (2021) Profiling the Tox21 Chemical Collection for Acetylcholinesterase Inhibition. Environ Health Perspect 129(4):47008 doi:10.1289/EHP6993

Lorendeau D, Rinaldi G, Boon R, et al. (2017) Dual loss of succinate dehydrogenase (SDH) and complex I activity is necessary to recapitulate the metabolic phenotype of SDH mutant tumors. Metab Eng 43(Pt B):187-197 doi:10.1016/j.ymben.2016.11.005

Marcker K, Sanger F (1964) N-Formyl-Methionyl-S-Rna. J Mol Biol 8:835-40 doi:10.1016/s0022-2836(64)80164-9

McGowan KA, Nyhan WL, Barshop BA, et al. (2004) The role of methionine in ethylmalonic encephalopathy with petechiae. Arch Neurol 61(4):570-4 doi:10.1001/archneur.61.4.570

Metzler M, Pfeiffer E (1995) Effects of estrogens on microtubule polymerization in vitro: correlation with estrogenicity. Environ Health Perspect 103 Suppl 7(Suppl 7):21-2 doi:10.1289/ehp.95103s721

Miller CP, Collini MD, Tran BD, et al. (2001) Design, synthesis, and preclinical characterization of novel, highly selective indole estrogens. J Med Chem 44(11):1654-7 doi:10.1021/jm010086m

Mullen AR, Wheaton WW, Jin ES, et al. (2011) Reductive carboxylation supports growth in tumour cells with defective mitochondria. Nature 481(7381):385-8 doi:10.1038/nature10642

Nyffeler J, Willis C, Lougee R, Richard A, Paul-Friedman K, Harrill JA (2020) Bioactivity screening of environmental chemicals using imaging-based high-throughput phenotypic profiling. Toxicol Appl Pharmacol 389:114876 doi:10.1016/j.taap.2019.114876

Olivieri G, Martinelli D, Longo D, et al. (2021) Ethylmalonic encephalopathy and liver transplantation: long-term outcome of the first treated patient. Orphanet J Rare Dis 16(1):229 doi:10.1186/s13023-021-01867-5

Pachnis P, Wu Z, Faubert B, et al. (2022) In vivo isotope tracing reveals a requirement for the electron transport chain in glucose and glutamine metabolism by tumors. Sci Adv 8(35):eabn9550 doi:10.1126/sciadv.abn9550

Pallag G, Nazarian S, Ravasz D, et al. (2022) Proline Oxidation Supports Mitochondrial ATP Production When Complex I Is Inhibited. Int J Mol Sci 23(9) doi:10.3390/ijms23095111

Purohit A, Radeke H, Azure M, et al. (2008) Synthesis and biological evaluation of pyridazinone analogues as potential cardiac positron emission tomography tracers. J Med Chem 51(10):2954-70 doi:10.1021/jm701443n

Rose L, Jenkins AT (2007) The effect of the ionophore valinomycin on biomimetic solid supported lipid DPPTE/EPC membranes. Bioelectrochemistry 70(2):387-93 doi:10.1016/j.bioelechem.2006.05.009

Ruetschi U, Cerone R, Perez-Cerda C, et al. (2000) Mutations in the 4-hydroxyphenylpyruvate dioxygenase gene (HPD) in patients with tyrosinemia type III. Hum Genet 106(6):654-62 doi:10.1007/s004390000307

Schildknecht S, Pape R, Meiser J, et al. (2015) Preferential Extracellular Generation of the Active Parkinsonian Toxin MPP+ by Transporter-Independent Export of the Intermediate MPDP+. Antioxid Redox Signal 23(13):1001-16 doi:10.1089/ars.2015.6297

Shi ZZ, Habib GM, Rhead WJ, et al. (1996) Mutations in the glutathione synthetase gene cause 5-oxoprolinuria. Nat Genet 14(3):361-5 doi:10.1038/ng1196-361

Smith AE, Marcker KA (1968) N-formylmethionyl transfer RNA in mitochondria from yeast and rat liver. J Mol Biol 38(2):241-3 doi:10.1016/0022-2836(68)90409-9

Sostare E, Lawson TN, Saunders LR, et al. (2022) Knowledge-Driven Approaches to Create the MTox700+ Metabolite Panel for Predicting Toxicity. Toxicol Sci 186(2):208-220 doi:10.1093/toxsci/kfac007

Srivastava P, Panda D (2007) Rotenone inhibits mammalian cell proliferation by inhibiting microtubule assembly through tubulin binding. FEBS J 274(18):4788-801 doi:10.1111/j.1742-4658.2007.06004.x

Suciu I, Delp J, Gutbier S, et al. (2023) Dynamic Metabolic and Transcriptional Responses of Proteasome-Inhibited Neurons. Antioxidants (Basel) 12(1) doi:10.3390/antiox12010164

Sullivan LB, Gui DY, Hosios AM, Bush LN, Freinkman E, Vander Heiden MG (2015) Supporting Aspartate Biosynthesis Is an Essential Function of Respiration in Proliferating Cells. Cell 162(3):552-63 doi:10.1016/j.cell.2015.07.017

Szewczyk A, Wojcik G, Nalecz MJ (1995) Potassium channel opener, RP 66471, induces membrane depolarization of rat liver mitochondria. Biochem Biophys Res Commun 207(1):126-32 doi:10.1006/bbrc.1995.1162

Terron A, Bal-Price A, Paini A, et al. (2018) An adverse outcome pathway for parkinsonian motor deficits associated with mitochondrial complex I inhibition. Arch Toxicol 92(1):41-82 doi:10.1007/s00204-017-2133-4

Thomopoulou P, Sachs J, Teusch N, Mariappan A, Gopalakrishnan J, Schmalz HG (2016) New Colchicine-Derived Triazoles and Their Influence on Cytotoxicity and Microtubule Morphology. ACS Med Chem Lett 7(2):188-91 doi:10.1021/acsmedchemlett.5b00418

Turner N, Li JY, Gosby A, et al. (2008) Berberine and its more biologically available derivative, dihydroberberine, inhibit mitochondrial respiratory complex I: a mechanism for the

action of berberine to activate AMP-activated protein kinase and improve insulin action. Diabetes 57(5):1414-8 doi:10.2337/db07-1552

van Vugt-Lussenburg BMA, van der Lee RB, Man HY, et al. (2018) Incorporation of metabolic enzymes to improve predictivity of reporter gene assay results for estrogenic and anti-androgenic activity. Reprod Toxicol 75:40-48 doi:10.1016/j.reprotox.2017.11.005

Vergara F, Plum F, Duffy TE (1974) Alpha-ketoglutaramate: increased concentrations in the cerebrospinal fluid of patients in hepatic coma. Science 183(4120):81-3 doi:10.1126/science.183.4120.81

Volbracht C, Leist M, Nicotera P (1999) ATP controls neuronal apoptosis triggered by microtubule breakdown or potassium deprivation. Mol Med 5(7):477-89

Waller JP (1963) The Nh2-Terminal Residues of the Proteins from Cell-Free Extracts of E. Coli. J Mol Biol 7:483-96 doi:10.1016/s0022-2836(63)80096-0

Wijaya LS, Rau C, Braun TS, et al. (2022) Stimulation of de novo glutathione synthesis by nitrofurantoin for enhanced resilience of hepatocytes. Cell Biol Toxicol 38(5):847-864 doi:10.1007/s10565-021-09610-3

Zhang B, Pan Y, Xu L, et al. (2018) Berberine promotes glucose uptake and inhibits gluconeogenesis by inhibiting deacetylase SIRT3. Endocrine 62(3):576-587 doi:10.1007/s12020-018-1689-y

Zorov DB, Juhaszova M, Sollott SJ (2014) Mitochondrial reactive oxygen species (ROS) and ROS-induced ROS release. Physiol Rev 94(3):909-50 doi:10.1152/physrev.00026.2013
